# *In vivo* and *in vitro* characterizations of melibiose permease (MelB) conformation-dependent nanobodies reveal sugar-binding mechanisms

**DOI:** 10.1016/j.jbc.2023.104967

**Published:** 2023-06-26

**Authors:** Satoshi Katsube, Katleen Willibal, Sangama Vemulapally, Parameswaran Hariharan, Elena Tikhonova, Els Pardon, H. Ronald Kaback, Jan Steyaert, Lan Guan

**Affiliations:** 1 Department of Cell Physiology and Molecular Biophysics, Center for Membrane Protein Research, School of Medicine, Texas Tech University Health Sciences Center, Lubbock, Texas, USA; 2 VIB Center for Structural Biology Research, VIB, Brussel, Belgium; 3 Structural Biology Brussels, Vrije Universiteit Brussel, Brussel, Belgium; 4 Department of Physiology and Department of Microbiology, Immunology, and Molecular Genetics, Molecular Biology Institute, University of California, Los Angeles, Los Angeles, California, USA

**Keywords:** Nanobody, conformational binder, protein-protein interactions, ITC, binding, sugar fermentations, two-hybrid assay, EIIA^Glc^

## Abstract

*Salmonella enterica* serovar Typhimurium melibiose permease (MelB_St_) is a prototype of the Na^+^-coupled major facilitator superfamily transporters, which are important for the cellular uptake of molecules including sugars and small drugs. Although the symport mechanisms have been well-studied, mechanisms of substrate binding and translocation remain enigmatic. We have previously determined the sugar-binding site of outward-facing MelB_St_ by crystallography. To obtain other key kinetic states, here we raised camelid single-domain nanobodies (Nbs) and carried out a screening against the WT MelB_St_ under 4 ligand conditions. We applied an *in vivo* cAMP-dependent two-hybrid assay to detect interactions of Nbs with MelB_St_ and melibiose transport assays to determine the effects on MelB_St_ functions. We found that all selected Nbs showed partial to complete inhibitions of MelB_St_ transport activities, confirming their intracellular interactions. A group of Nbs (714, 725, and 733) was purified, and isothermal titration calorimetry measurements showed that their binding affinities were significantly inhibited by the substrate melibiose. When titrating melibiose to the MelB_St_/Nb complexes, Nb also inhibited the sugar-binding. However, the Nb733/MelB_St_ complex retained binding to the coupling cation Na^+^ and also to the regulatory enzyme EIIA^Glc^ of the glucose-specific phosphoenolpyruvate/sugar phosphotransferase system. Further, EIIA^Glc^/MelB_St_ complex also retained binding to Nb733 and formed a stable supercomplex. All data indicated that MelB_St_ trapped by Nbs retained its physiological functions and the trapped conformation is similar to that bound by the physiological regulator EIIA^Glc^. Therefore, these conformational Nbs can be useful tools for further structural, functional, and conformational analyses.

Nanobodies (Nbs) are the variable domains of single polypeptide heavy-chain antibodies from camelid mammals such as camels and llamas (1). Nbs are as small as one-tenth of common antibodies and are characteristic of longer complementarity-determining region 3 (CDR-3) in length (2). Their finger-like motifs can recognize cleft or hidden epitopes unavailable to monoclonal antibodies. They are known to exhibit high specificity and affinity. Conformation-specific Nbs can trap the membrane transport proteins of interest at high-energy states by binding to epitopes that might be transiently available. Nbs have been successfully applied for structural and functional studies of many membrane proteins (3, 4, 5, 6, 7).

Major facilitator superfamily (MFS) transporters are widely expressed from bacteria to mammalian cells and play important roles in health and disease (8). The MFS transporters contain a large group of cation-coupled symporters or antiporters. The melibiose permease of *Salmonella* enterica serovar Typhimurium (MelB_St_) catalyzes the symport of a galactopyranoside with a cation (H^+^, Li^+^, or Na^+^). It is a prototype for Na^+^-coupled MFS transporters and serves as a model system for studying cation-coupled transport mechanisms (9, 10, 11, 12, 13, 14). The computational predictions (15, 16) and high-resolution 3D X-ray crystal structure determination (10, 14) are consistent with the canonical MFS fold. The crystal structures with a bound sugar analog are also determined, which reveals the sugar specificity determinant pocket and the sugar recognition mechanism (14). MelB_St_ in all solved X-ray crystal structures is at an outward-facing conformation; the substrate-binding site is only accessible from the periplasmic side (Fig. 1). The presence of a physical barrier called inner barrier at this conformation prevents the substrate from moving into the cytoplasm (Fig. 1*A*).

**Figure 1:**
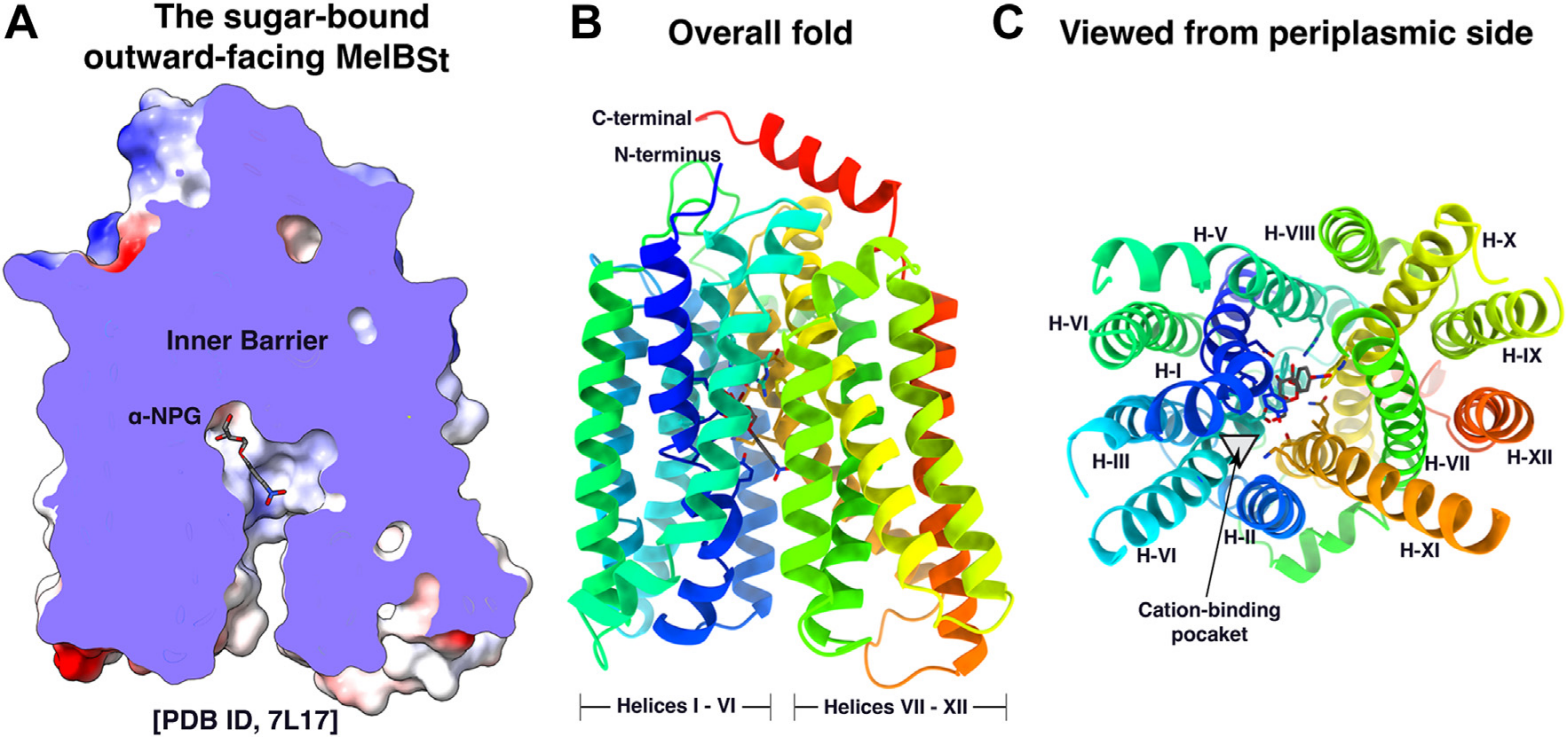
**Crystal structure of the D59C uniporter MelB**_**St**_**mutant with a sugar bound [PDB ID, 7L17].** MelB_St_ exhibits a canonical MFS fold. *A*, the sugar-bound outward-facing MelB_St_ as shown by a cross-section of the surface representation. The sugar substrate α-nitrophenyl galactoside (α-NPG) highlighted in *dark gray* is bound at the apex of the outward-open cavity, and its access to the cytoplasm is blocked by the thick inner barrier. *B*, overall fold, the transmembrane helices I-VI and VII-XII form two helix bundles and the cytoplasmic ends are closed that is stabilized by the C-terminal tail helix. Molecular α-NPG and the residues involved in its binding are highlighted in the *sticks*. *C*, viewed from the periplasmic side. The transmembrane helices were labeled in *Roman numerals*. The cation-binding pocket is indicated by a *pink triangle*. The D59C uniporter mutant does not bind a cation. MelB_St,_*Salmonella enterica* serovar Typhimurium melibiose permease; MFS, major facilitator superfamily; Nbs, nanobodies; PDB, Protein Data Bank.

It is well-known that the MFS transporters should change their conformation to allow the substrate translocation from one side of the membrane to the other. To analyze these structurally unresolved states of melibiose permease (MelB), 10 Nbs were raised and selected from the WT MelB_St_. To determine the binding sidedness of all Nbs with the membrane protein MelB_St_, we adapted a bacterial adenylate cyclase (CyaA)-based two-hybrid system, which has been widely used for soluble protein-protein interaction studies (17, 18, 19, 20, 21); however, this method has not been well explored for soluble-membrane protein interaction studies yet. This assay relies on two hybrid proteins containing the T18 and T25 fragments of the adenylate domain of a *Bordetella pertussis* toxin (Fig. 2*A*). Co-expression of both fragments in *Escherichia coli* cells does not generate CyaA activity unless both are fused with a pair of interacting proteins (17, 22). Nb and MelB_St_ interactions were examined by this *in vivo* assay.

**Figure 2:**
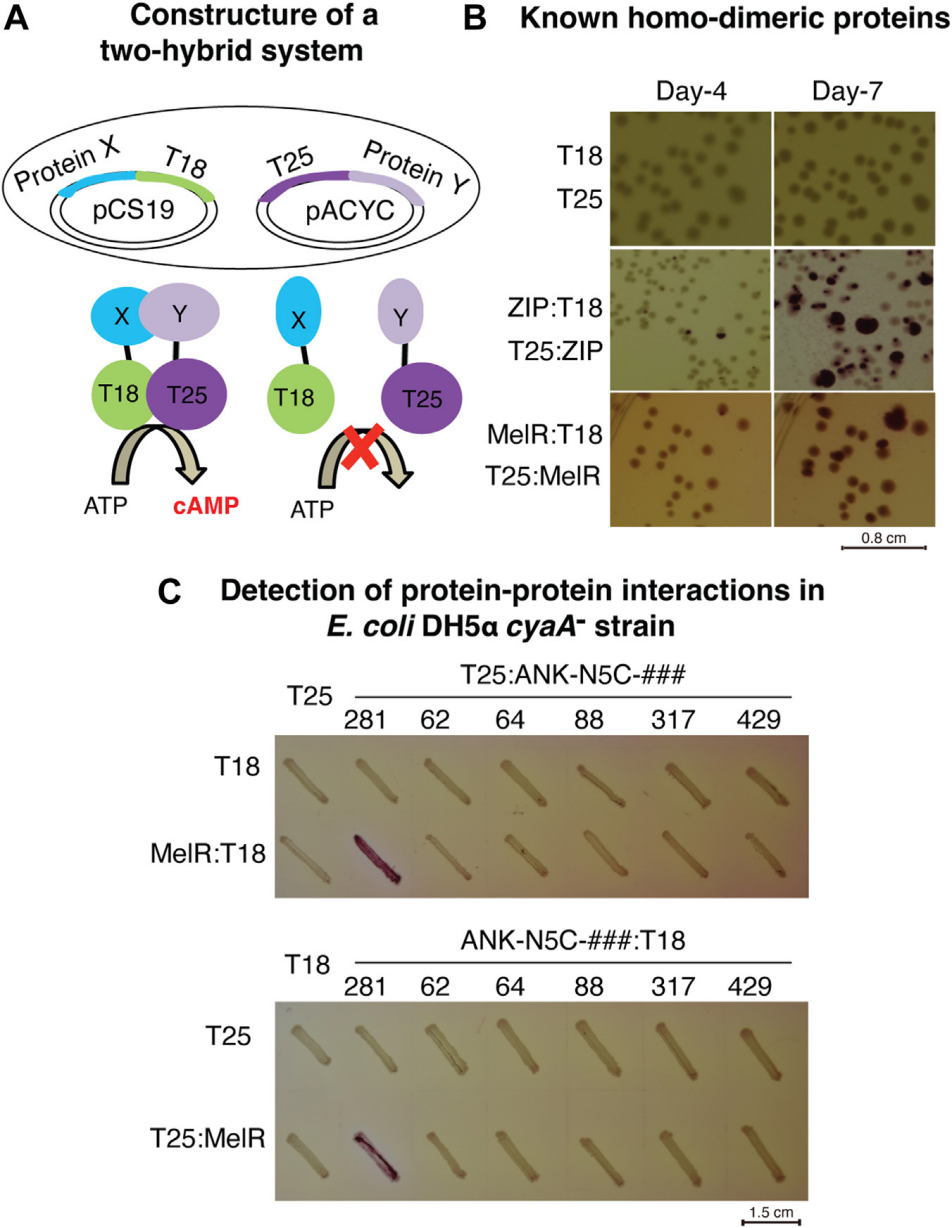
**Two-hybrid assay with *Escherichia coli* DH5α *ΔcyaA* strain.***A*, a scheme for adenylate cyclase-based two-hybrid system. When the T18 and T25 fragments are expressed separately, no cAMP is produced. The interaction of two hybrid proteins with X and Y brings out the T18 and T25 fragments together and restores adenylate cyclase activities and cAMP production. The framed images in *blue color* for the two hybrid results containing the Nb732:T18 fusion and Nb738:T18 fusion were obtained separately due to the slow development of the *red color*. *B*, DNA-binding leucine zipper (ZIP) and a transcription factor MelR were fused to T18 and T25 vectors, respectively. Co-expression encoded by two compatible vectors was carried out in a created DH5α *ΔcyaA* strain (Table S1) and incubated on MacConkey agar plates containing 30 mM maltose as the sole carbohydrate source. The pictures were taken after 4 days or 7 days of incubation at 30 °C. *C*, interaction of MelR and ANK-N5C-281. Two polarities of fusions were tested. The plasmids encoding T25:ANK-N5C fusion (ANK-N5C was fused to the C terminus of T25 fragment) and ANK-N5C:T18 fusion (ANK-N5C was fused to the N terminus of T18 fragment) were individually co-transformed with a compatible vector pCS19/MelR-T18 or pACYC/T25:MelR into the *cyaA*^-^ DH5α cells. The results are shown in patching from a single colony on the MacConkey agar plates containing 30 mM maltose. Only those cells containing a pair of fusions with ANK-N5C-281 and MelR restored the maltose fermentation in the *ΔcyaA* strain. CyaA, adenylate cyclase.

We also performed extensive *in vivo* and *in vitro* characterizations of Nbs binding and effects on MelB_St_ functions, including transport activities, binding affinities for substates melibiose, Na^+^, and the regulatory enzyme EIIA^Glc^ in the glucose-specific phosphoenolpyruvate/sugar phosphotransferase system (23, 24, 25). Few Nbs have been identified to trap MelB at a possible Na^+^-bound and sugar low-affinity conformation. Those Nbs could serve as a useful tool for structural analysis.

## Results

### Generation of MelB_St_ Nbs

The WT MelB_St_ proteins were purified and functionally reconstituted into proteoliposomes as described (13), and llama immunization and library construction were also described (1). The selections by phage display using WT MelB_St_ proteoliposomes were performed under 4 conditions (Na^+^ or Li^+^ in the absence or presence of melibiose). A total of 11 representative Nbs (*ca.* 126–132 amino acid residues) were obtained (Table 1), which can be grouped into 6 groups based on the amino acid sequence similarity in the CDR-3. It is likely that Nb from the same group might bind to the same target epitope. Two Nbs CA10718 and CA10833 (the short ID Nb718 and Nb733), which were obtained from 2 different selection conditions, are silent variants with identical amino acid sequences. Thus, 10 Nbs with unique amino acid sequences were collected for further studies. The group 1 were found from all 4 selection conditions with a low occurrence (5 times), and group 4 members were obtained from both melibiose-containing conditions or Li^+^ alone, with a high occurrence (99 times from each condition). The selection results indicate that MelB_St_ bound with either cation Na^+^ or Li^+^ alone or together with melibiose could generate a similar epitope to be recognized by the group 1 Nbs even with a low occurrence.

**Table 1:** Nb selection

Reference No.	Nb ID No.	Group	Occurrence^a^	Selection condition	Inhibition on melibiose active transport	Binding site
CA10714	Nb714	1	5	Li^+^	Full inhibition	Cytoplasmic side
CA10718	Nb718^b^		5	Li^+^ and melibiose	Full inhibition	Cytoplasmic side
CA10725	Nb725		5	Na^+^	Full inhibition	Cytoplasmic side
CA10733	Nb733^b^		5	Na^+^ and melibiose	Full inhibition	Cytoplasmic side
CA10732	Nb732	2	2	Na^+^ and melibiose	Partial inhibition	Cytoplasmic side
CA10721	Nb721	3	17	Na^+^	Full inhibition	Cytoplasmic side
CA10712	Nb712	4	99	Li^+^	Partial inhibition	Cytoplasmic side
CA10715	Nb715		99	Li^+^ and melibiose	Partial inhibition	Cytoplasmic side
CA10728	Nb728		99	Na^+^ and melibiose	Partial inhibition	Cytoplasmic side
CA10738	Nb738	5	6	Na^+^ and melibiose	Partial inhibition	Cytoplasmic side
CA10723	Nb723	6	2	Na^+^	Full inhibition	Cytoplasmic side

a The number of group members under each selection condition was obtained.

b Identical amino acid sequence but different at one nucleobase position.

### *In vivo* protein-protein interactions analyzed by a bacterial two-hybrid assay

To analyze intracellular protein-protein interactions, a bacterial two-hybrid assay based on CyaA toxin T18/T25 fragments of *B. pertussis* was established. Three *cyaA* gene-deleted *E. coli* strains including DH5α, DW2, and T7 Express were created (Table S1; Fig. 2*A*). In *E. coli*, cAMP was required for these cAMP-dependent sugar utilizations, such as maltose, melibiose, lactose, and so on. To assess the *cyaA*^*-*^ phenotype, the cAMP-dependent sugar fermentations were carried out on MacConkey agar plates containing maltose or melibiose as a sole carbohydrate source. Two of the parent strains DH5α and T7 Express fermented both sugars, as shown by the red-forming colonies after incubation overnight (Fig. S1*A*). The DW2 strain (*mel*A^+^, *melB*^*-*^, and *lacZ*^*-*^*Y*^*-*^) only fermented maltose, not melibiose, since lacking melibiose transport, so grew as yellow colonies (26).

The created DH5α *cyaA*^*-*^ strain grew slowly and formed yellow colonies on the melibiose plates after 18-h incubation at 30 °C (Fig. S1*A*); interestingly, the colonies grown on the maltose plates showed transient red for 3 to 4 days after the transformation, and the color eventually changed to yellow with further incubation. The results indicated no further maltose utilization, which is consistent with the lack of cAMP production due to the *cyaA* gene deletion. With the supplement of 0.5 mM cAMP into the media, the *cyaA*^*-*^ strain formed red colonies on both sugar plates after 2 to 3 days and maintained the red color for at least 10 days. The T7 Express *cyaA*^*-*^ strain showed similar phenotypes with more robust fermentation of both sugars. The DW2 *cyaA*^*-*^ strain also exhibited cAMP-dependent maltose fermentation but had no melibiose fermentation in the absence or presence of supplement cAMP as expected.

The disaccharide utilization by the DH5α *cyaA* strain was further tested by monitoring the cell-growth curves in M9 minimal media containing melibiose or maltose as the sole carbon source. The *cyaA*^-^ cells did not grow on either sugar (Fig. S1*B*) unless 0.5 mM cAMP was added into the media; even then, the growth rate was much slower than that of the WT in the absence or presence of cAMP. All data support the conclusion that the *cyaA* gene-deleted mutants lost the capability to utilize either melibiose or maltose due to the lack of cAMP production, which agrees with the previous reports (17, 18, 22).

To verify the two-hybrid interaction assay, 2 DNA-binding dimeric proteins leucine zipper (*ZIP*) (22) and the transcription activator of melibiose operon *melAB* in *E. coli* (MelR) (27) were used as the positive control. Two pairs of compatible fusion plasmids encoding 2 ZIP (or 2 MelR) fused with T18 and T25, respectively, were co-transformed into the DH5α *cyaA* strain (Fig. 2*B*). As described for another indicator strain (17, 18), the cells formed small colonies after overnight incubation on the maltose MacConkey agar plates at 30 °C. For the ZIP:T18 and T25:ZIP hybrids, or MelR:T18 and T25:MelR hybrids, few red cells showed up after a 4 to 5 incubation. These red colonies became larger with irregular shapes and maintained the color for at least 10 days, which indicated the homodimeric formation of ZIP or MelR proteins, respectively. The protein-protein interactions of either monomeric ZIP or MelR facilitated the interactions of T18 with T25 fragments, reconstituting the CyaA activities. As the negative control, the cells expressing T18 and T25 fragments without a fusion, or fused with only the ZIP or MelR, appeared yellow. The colony’s color remained longer even after incubation for more than 10 days. The final results, which were represented in patches including varying negative controls (Fig. S1*C*), validated this cAMP-dependent two-hybrid assay.

A previously created transcription inhibitor ankyrin ANK-N5C-281 by a combinatorial approach has been proposed to interact with MelR and inhibit its function, but direct evidence of their interaction was missing (27). The two-hybrid assay supported the interactions of the ANK-N5C-281 with MelR regardless of the polarity of fusion (Fig. 2*C*). Five other ankyrin N5C proteins (62, 64, 88, 317, or 429), which are not the *mel* operon inhibitor, showed no interaction with MelR (27). This study provided evidence for the direct interaction of N5C-281 and MelR.

### Intracellular interactions of Nbs with MelB_St_

**Figure 3:**
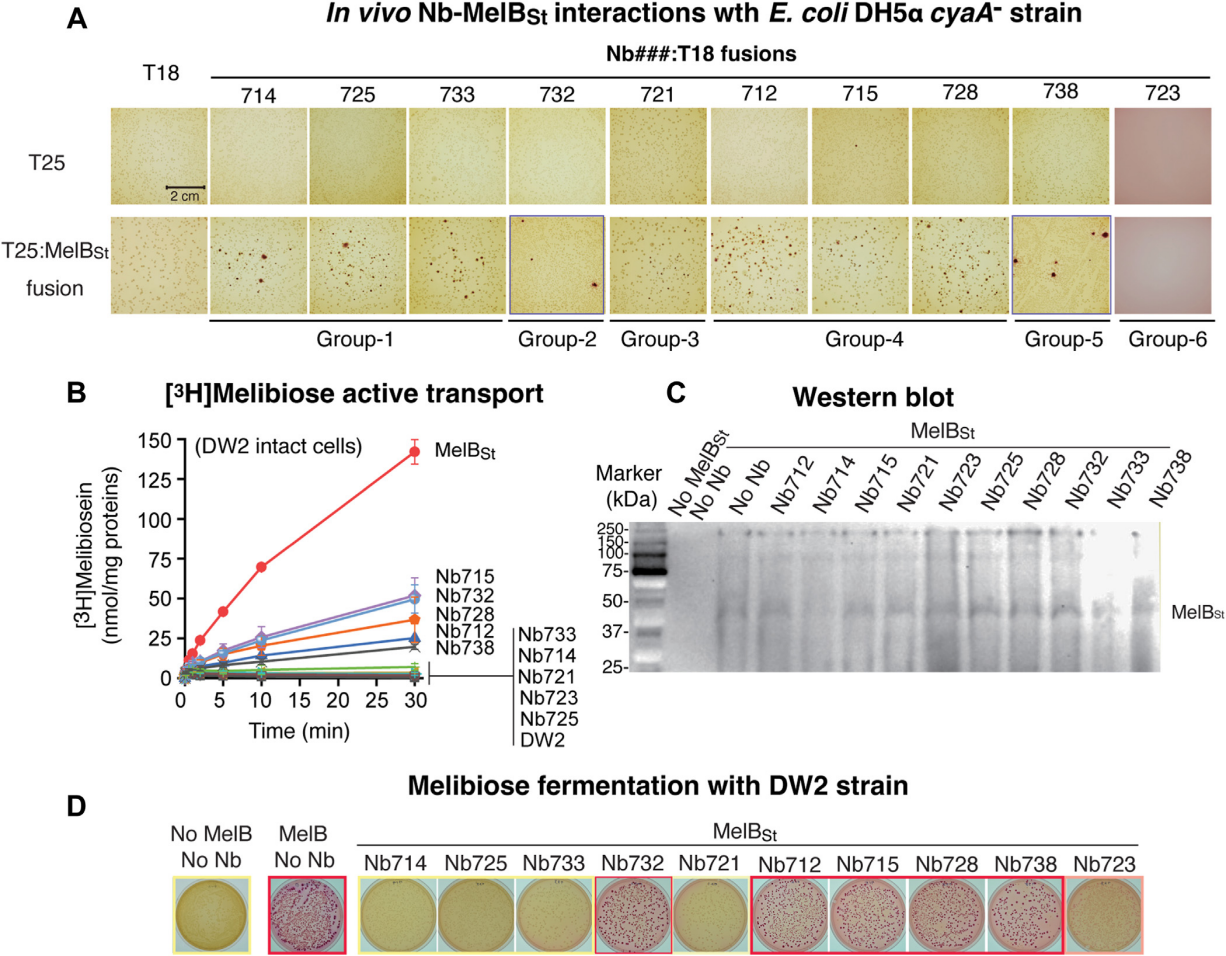
***In vivo* Nbs binding and effect on MelB transport activities.***A*, *in vivo* cAMP-based two-hybrid assay. Each Nb was fused to the N-terminus of the T18 fragment on the plasmid pCS19/X:T18/FX vector to generate the Nbs:T18 fusions, and MelB_St_ was fused to the C terminus of the T25 on the plasmid pACYC/T25:X/FX to generate the T25:MelB_St_ fusion. The 2 types of compatible plasmids containing the T18 (labeled on the *top*) and T25 (labeled at the *left side*) without or with a fusion were co-transformed at all possible combinations into the indicator strain *Escherichia coli* DH5α *ΔcyaA* strain. The cells were grown on maltose-containing MacConkey agar plates as described in the Experimental procedures. The picture was taken after a 7-day incubation at 30 °C. *Red* colonies indicate a positive result. *B*, inhibition of [^3^H]]melibiose active transport. *E. coli* DW2 (*melB*^*-*^*lacY*^*-*^*Z*^*-*^) was transformed with 2 compatible expression vectors pACYC/MelB_St_ and pCS19/Nbs, encoding WT MelB_St_ and Nbs (without any fusion), respectively. With intact cells, the Na^+^-coupled melibiose uptake was stopped at the indicated time points. Error bar, SEM; the number of tests = 2 to 3. The DW2 cells containing the pACYC/MelB_St_ and pCS19 without Nb were used as the positive control, and pACYC and pCS19 with no MelB nor Nb were used as the negative control. *C*, Western blot, membranes expressing MelB_St_ alone or co-expressed with Nbs in a buffer containing 50 mM NaPi, pH 7.5, 100 mM NaCl, and 10% glycerol were prepared as described in Experimental procedures. An aliquot of 50 μg membrane proteins of each sample was analyzed by SDS-15%PAGE, and MelB_St_ proteins were detected by Western blotting using anti-His tag antibody as described in Experimental procedures. Control, membranes isolated from the DW2 cells carrying the 2 empty vectors pCS19 and pACYC with no protein. *D*, inhibition of melibiose fermentation. *E. coli* DW2 was transformed with pACYC/MelB_St_ and pCS19/Nbs encoding the WT MelB_St_ and an Nb without any fusion, respectively. The cells with pACYC/MelB_St_ and pCS19 were used as the positive control, and empty vectors, pACYC and pCS1, were used as the negative control. MacConkey agar plates containing 30 mM melibiose as the sole carbohydrate source were incubated at 30 °C, and photos were taken after 18 h incubation. This conventional melibiose fermentation assay detects the melibiose transport rate that limits the fermentation rate and the degree of acidification as indicated by the *color shades*. *Red* colonies, normal melibiose fermentation; *yellow* colonies, no fermentation; and *pink* colonies, reduced fermentation rates. CyaA, adenylate cyclase; MelB_St,_*Salmonella enterica* serovar Typhimurium melibiose permease; Nbs, nanobodies.

The 10 Nbs were individually fused to the N-terminus of T18 and then transformed into the DH5α *cyaA*^-^ cells pre-transformed with a compatible plasmid carrying T25:MelB_St_ (Table S1). All, except the Nb723:T18 fusion, showed positive maltose fermentation as indicated by the appearance of red colonies (Fig. 3*A*). There was no colony growth on the plates containing the Nb723:T18 fusion. The results indicated that at least 9 Nbs bind to the cytoplasmic side of MelB_St,_ which validated this method for soluble and membrane protein interactions.

### Effects of Nbs on MelB_St_ transport activity

*E. coli* DW2 cells (*mel*A^+^, *melB*^*-*^, and *lacZ*^*-*^*Y*^*-*^), which were used for the expression and functional characterization of MelB, were transformed with 2 compatible expression plasmids encoding for MelB_St_ and the Nbs, respectively, and subjected to the Na^+^-coupled [^3^H]melibiose active transport assay against melibiose concentration gradient as described in Methods (26). Compared with the WT MelB_St_, all Nbs inhibited the melibiose active transport activities to varying extents (Fig. 3*B*). Five Nbs (Nbs714, 725, 733, 721, and 723) nearly inactivated MelB_St_, and the other 5 Nbs (Nb712, 715, 728, 732, and 738) showed 60 to 85% inhibitions. The Western blot of membranes prepared from the cells co-expressing both MelB_St_ alone or with Nbs showed that MelB_St_ protein expression when co-expressed with each individual Nb is comparable to the WT MelB_St_ proteins. However, the expression levels were very low (Fig. 3*C*).

The melibiose fermentation assay with DW2 (*lacY*^-^*melB*^*-*^), which was routinely used to report the melibiose down-concentration transport activities, was also applied to test the effects of intracellular expressed Nbs on the co-expressed MelB_St_ (Fig. 3*D*). Out of the 5 Nbs (Nbs714, 725, 733, 721, 723) that completely inhibited the MelB_St_ active transport, 4 also completely inhibited melibiose fermentation and one (Nb723) showed partial inhibition since the colonies were pink. The other 5 Nbs with partial inhibitions on active transport showed little or no inhibition of melibiose fermentation. While each of the Nbs binds to the cytoplasmic side of MelB_St_, the inhibition of the MelB_St_ function varied.

### Nb binding and substrate effects determined by ITC

**Figure 4:**
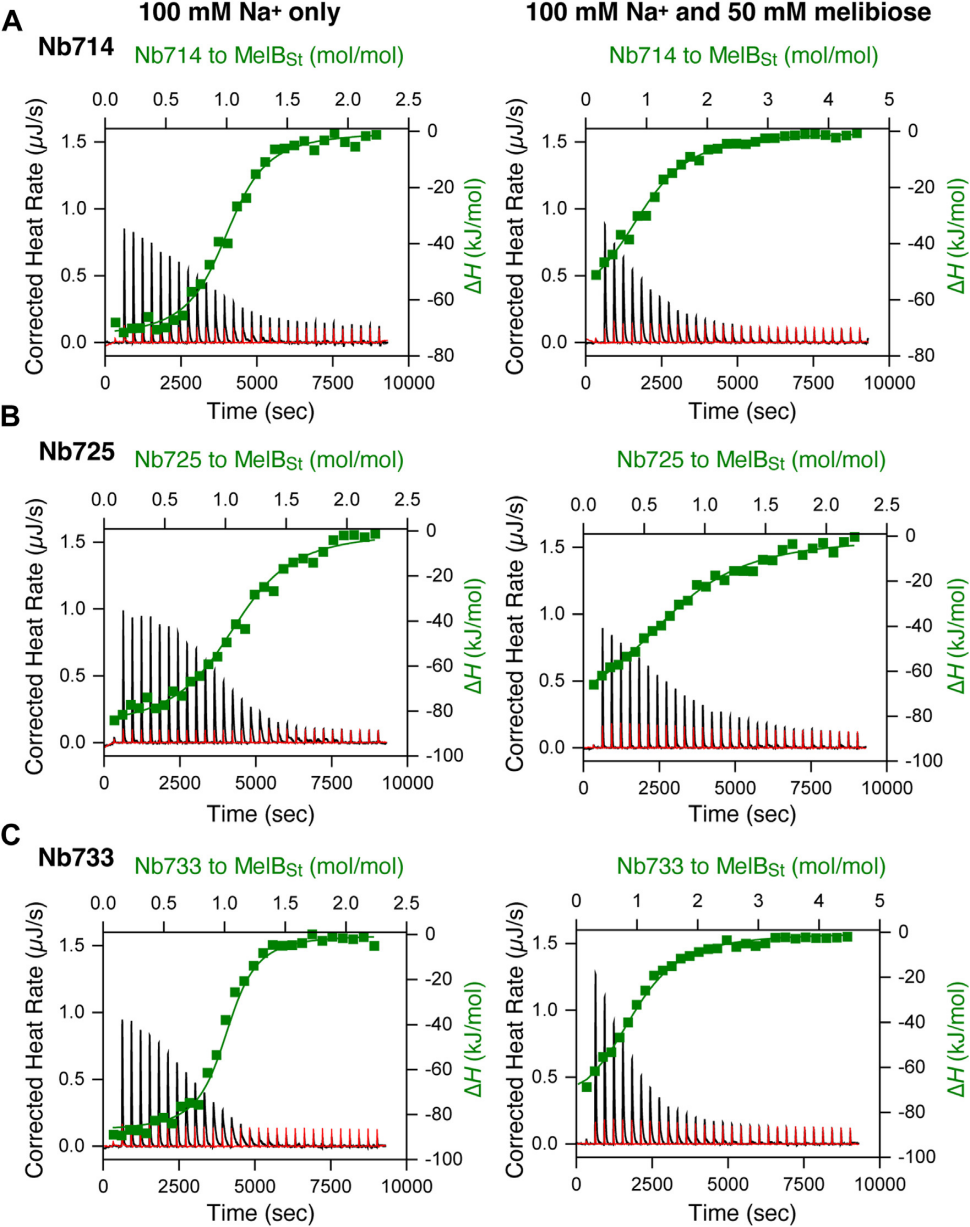
**Measurements of Nbs binding to MelB**_**St**_**and melibiose effect by ITC.** Nbs binding to the Na^+^-bound WT MelB_St_ in the absence or presence of 50 mM melibiose was collected with ITC calorimeters (TA Instruments) at 25 °C. For each measurement, an aliquot of 30 μM of MelB_St_ in the buffer of 20 mM Tris–HCl, pH 7.5, 100 mM NaCl, 0.01% DDM, and 10% glycerol without or with the addition of 50 mM melibiose was placed in the sample cell. Solutions containing Nbs of 180 to 200 μM were prepared in a matching buffer, and placed in the syringe, and incrementally injected with 2 μl aliquots into the sample cell at 300-s intervals as described in the Experimental procedures. *A*, Nb714 binding to WT MelB_St_. *B*, Nb725 binding to WT MelB_St_. *C*, Nb733 binding to WT MelB_St_. The thermogram was plotted as the baseline-corrected heat rate (μJ/s; *left* axis) *versus* time (*bottom* axis) for the titrant to MelB_St_ (*black*) or to buffer (*red*) under an identical scale. The enthalpy change (Δ*H*) (kJ/mol; *filled green symbol*) was plotted against the Nb/MelB_St_ molar ratio (*top*/*right* axes). DDM, n-dodecyl-β-D-maltopyranoside; ITC, isothermal titration calorimetry; MelB_St,_*Salmonella enterica* serovar Typhimurium melibiose permease; Nbs, nanobodies.

All Nbs were subjected to cytoplasmic expression and metal affinity purification. Only 3 Nbs from group 1 (Nbs714, 725, and 733), which inhibited both active transport and melibiose fermentation, can be concentrated for isothermal titration calorimetry (ITC) measurements to determine the dissociation constants (*K*_d_) (Fig. 4; Table 2).

**Table 2:** Nb binding thermodynamics

Syringe	Sample cell	*K*_d_ (μM)	Δ*G* (kJ/mol)	Δ*H* (kJ/mol)	-*T*Δ*S* (kJ/mol)	*N*	Increase in *K*_d_	Decrease in Δ*H* (kJ/mol)
Nb714	MelB_St_/Na^+^	0.69 ± 0.11^a^	−35.21 ± 0.40	−78.83 ± 4.52	43.63 ± 4.13	0.92 ± 0.09	9.2-fold	6.11
	MelB_St_/Na^+^/Mel	6.35 ± 0.42	−29.69 ± 0.42	−72.72 ± 6.46	43.03 ± 6.28	0.96 ± 0.03		
Nb725	MelB_St_/Na^+^	1.61 ± 0.08	−33.08 ± 0.13	−88.24 ± 0.84	55.16 ± 20.72	1.01 ± 0.09	4.08-fold	4.27
	MelB_St_/Na^+^/Mel	6.56 ± 0.83	−29.61 ± 0.31	−83.97 ± 1.42	54.35 ± 0.06	0.85 ± 0.06		
Nb733	MelB_St_/Na^+^	0.52 ± 0.07	−35.90 ± 0.32	−89.92 ± 2.64	54.01 ± 2.97	0.98 ± 0.03	11.73-fold	4.78
	MelB_St_/Na^+^/Mel	6.10 ± 0.03	−29.77 ± 0.01	−85.14 ± 3.67	55.38 ± 3.68	0.98 ± 0.03		

a Error bar, SEM; the number of tests = 2 for all.

The binding of the Nbs to MelB_St_ in the presence of Na^+^, or Na^+^ and melibiose, released heat, showing exothermic thermographs. In the absence of the melibiose, the curve shape for the binding exhibited a near-ideal sigmoidal feature and can be fitted to stoichiometric numbers close to 1. The *K*_d_ values for Nb714, Nb725, and Nb733 in the presence of Na^+^ were 0.69 ± 0.11, 1.61 ± 0.08, and 0.52 ± 0.07 μM, respectively. In either case, the higher binding enthalpy (Δ*H*) made the sole favorable contribution to the binding free energy (Δ*G*) after compensating for the unfavorable large entropic changes. The Nbs/MelB_St_ protein interactions were also supported by gel-filtration chromatography (Fig. S2). The Nb733 and MelB_St_ migrate differently at 13.02 ml and 16.66 ml, respectively (Fig. S2*A* upper panel). When mixed with MelB_St_, Nb733 results in a slightly left shift by 0.11 ml of the MelB_St_ peak (Fig. S2*A* upper panel).

The effects of MelB substrates (melibiose and Na^+^) on the Nb binding were also carried out (Fig. 4; Table 2). In the presence of both melibiose and Na^+^, all of the 3 binding affinities were significantly reduced by approximately 10-fold for Nbs714 and 733 and by 4-fold for Nb725. The decrease in the binding enthalpy contributed to the major affinity decrease. These results indicated that the sugar substrate inhibits the Nbs binding.

### Differential effects of Nbs on MelB_St_ affinities to melibiose and Na^+^

**Figure 5:**
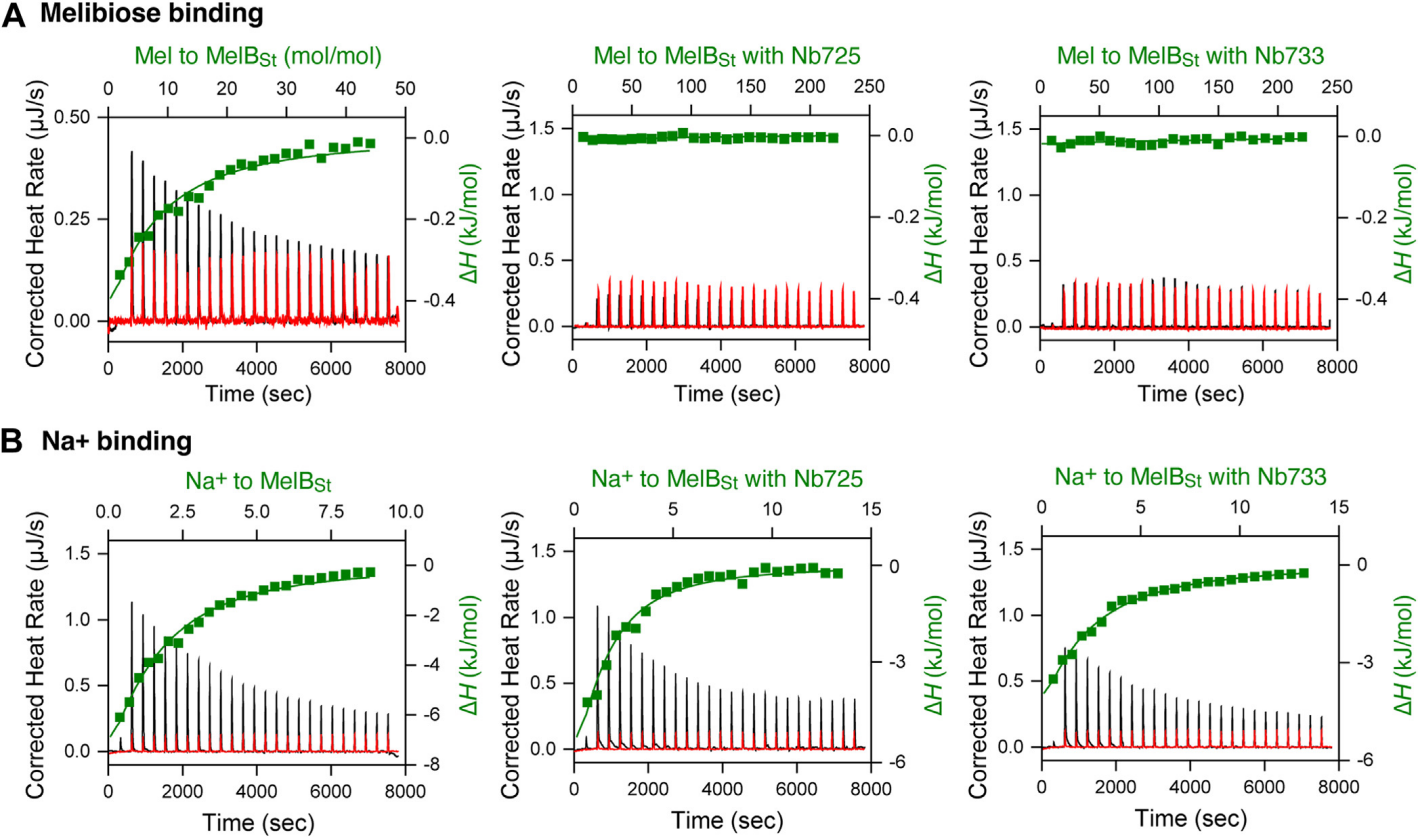
**Effect of Nbs on melibiose and Na**^**+**^**binding to MelB**_**St**_**by ITC.** Melibiose or Na^+^ binding to Nb-bound or free WT MelB_St_ was collected with ITC calorimeters (TA Instruments) at 25 °C. For each measurement, an aliquot of 80 μM of MelB_St_ without or with an Nb (725 or 733) at a concentration of 120 μM (molar ratio of 1:1.5) in the buffer of 20 mM Tris–HCl, pH 7.5, 50 mM choline chloride, 0.01% DDM, and 10% glycerol was placed in the sample cell. Solutions containing melibiose (10 mM or 50 mM free MelB_St_ or MelB_St_/Nb733 complex, respectively), or Na^+^ (5 or 2 mM for free MelB_St_ or MelB_St_/Nb733 complex, respectively) were prepared in a matching buffer and placed in the syringe and incrementally injected in 2 μl aliquots into the sample cell. *A*, melibiose binding to free WT MelB_St_ or complexed with Nb725 or Nb733. *B*, Na^+^ binding to free WT MelB_St_ or complexed with Nb725 or Nb733. The thermogram was plotted as the baseline-corrected heat rate (μJ/s; *left* axis) *versus* time (*bottom* axis) for the titrant to MelB_St_ or MelB/Nb complex (*black*) or to buffer (*red*) under an identical scale. Δ*H* (kJ/mol; filled *green symbol*) was plotted against the Nb/MelB_St_ molar ratio based on the *top*/*right* axes. DDM, n-dodecyl-β-D-maltopyranoside; ITC, isothermal titration calorimetry; Mel, melibiose; MelB_St,_*Salmonella enterica* serovar Typhimurium melibiose permease; Nbs, nanobodies.

Nb714, 725, and 733 completely inhibited melibiose transport activity when co-expressed with MelB_St_ (Fig. 3, *A* and *C*). The Nb733 and Nb714 only differ in one side chain, and Nb733 and Nb725 have further been analyzed for their effects on MelB binding affinities to melibiose and Na^+^ (Fig. 5; Table 3). In the presence of Na^+^, melibiose binding to MelB_St_ in n-dodecyl-β-D-maltopyranoside (DDM) buffer exhibits *K*_d_ of 1.43 ± 0.03 mM, which is slightly higher than that with MelB_St_ in undecyl-β-D-maltopyranoside (UDM) buffer (12, 24, 28). Surprisingly, when performing melibiose binding to the MelB_St_ complexed with Nb725 or Nb733 in the presence of Na^+^, even with an increased sugar concentration, both the peak height and change pattern are indistinguishable from that derived from the injection of melibiose at the same concentration into the buffer without a protein (Fig. 5).

**Table 3:** Nb effects on MelB_St_ binding for melibiose or Na^+^

Syringe	Sample cell	*K*_d_ (mM)	Δ*G* (kJ/mol)	Δ*H* (kJ/mol)	-*T*Δ*S* (kJ/mol)	*N*
Melibiose	MelB_St_/Na^+^	1.43 ± 0.03^a^	−16.23 ± 0.04	−6.95 ± 0.64	−9.28 ± 0.69	1
	MelB_St_/Na^+^/Nb725	/b	/	/	/	/
	MelB_St_/Na^+^/Nb733	/	/	/	/	/
Na^+^	MelB_St_	0.17 ± 0.02	−21.15 ± 0.36	−22.12 ± 2.06	0.61 ± 2.42	1
	MelB_St_/Nb725	0.18 ± 0.01	−21.44 ± 0.14	−16.14 ± 0.28	−5.3 ± 0.13	1
	MelB_St_/Nb733	0.13 ± 0.01	−22.22 ± 0.36	−17.00 ± 3.52	−5.21 ± 3.87	1
	MelB_St_/EIIA^Glc^^c^	0.25 ± 0.01	−20.53 ± 0.11	−31.54 ± 2.96	11.01 ± 2.85	1
EIIA^Glc^	MelB_St_	3.33 ± 0.49	−31.31 ± 0.45	−39.93 ± 2.97	8.62 ± 2.51	0.81 ± 0.01
	MelB_St_/Nb733	8.12 ± 0.14	−29.06 ± 0.04	−28.23 ± 0.34	−0.83 ± 0.30	0.76 ± 0.04
Nb733	MelB_St_	0.52 ± 0.07	−35.90 ± 0.32	−89.92 ± 2.64	54.01 ± 2.97	0.98 ± 0.03
	MelB_St_/EIIA^Glc^	0.75 ± 0.06	−34.98 ± 0.02	−40.22 ± 3.04	5.25 ± 3.05	0.98 ± 0.03

a Error bar, SEM; the number of tests = 2 for all.

b Not detectable.

c Detergent in the buffer in this assay is UDM.

Interestingly, Na^+^ binding to MelB_St_ in DDM buffer exhibits *K*_d_ of 1.17 ± 0.02 mM, slightly lower than that with MelB_St_ in UDM buffer (12, 24, 28). When titrating Na^+^ to MelB_St_/Nb725 or Nb733 complexes, the *K*_d_ values are indistinguishable from the binding with the free MelB_St_, 0.18 ± 0.02 or 0.13 ± 0.01 μM, respectively (Table 3). The entropic components contributed favorably to the binding-free energy and compensated for the reduced enthalpic change.

### Effects of Nb733 on MelB_St_ binding to its physiological binder EIIA^Glc^

**Figure 6:**
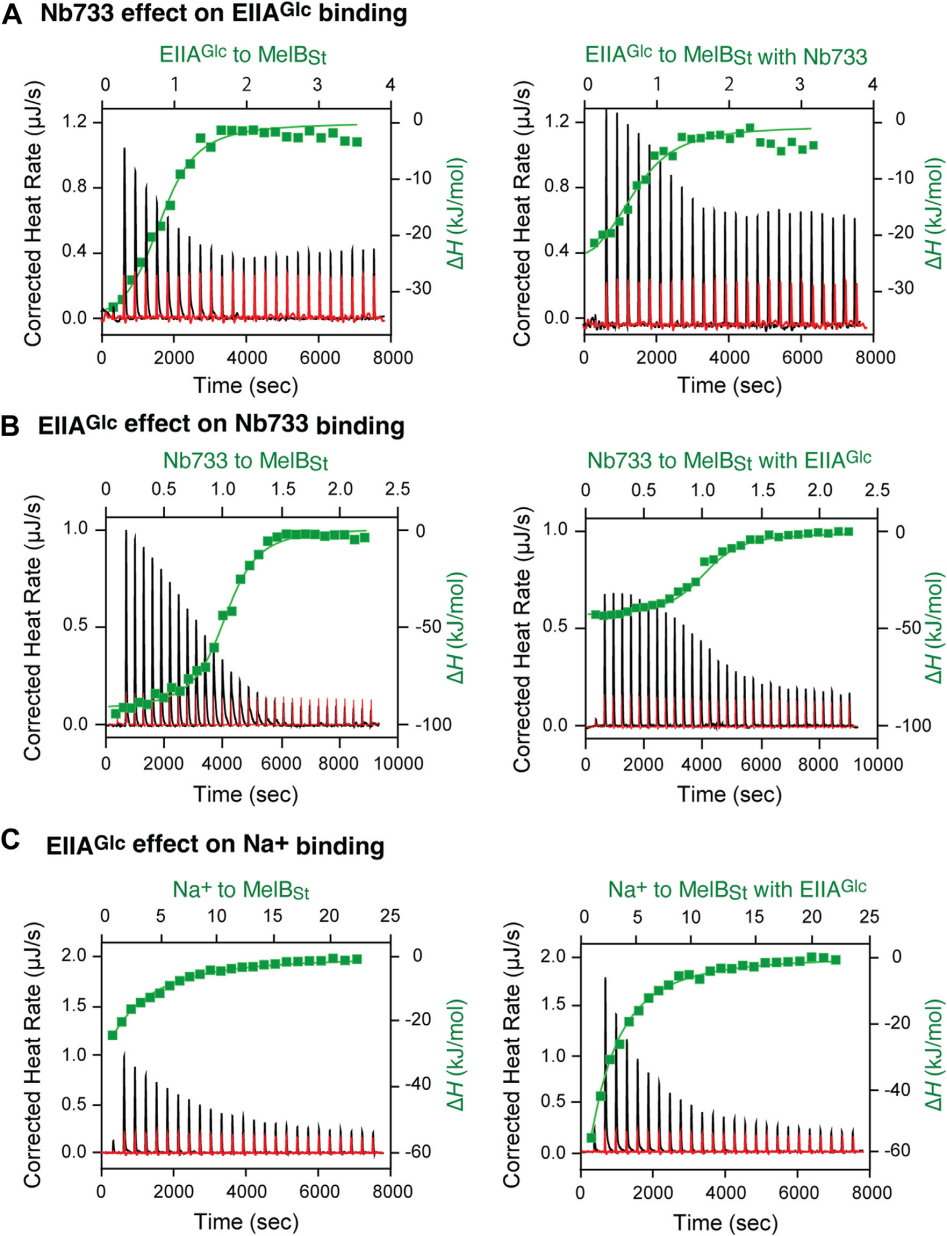
**Regulatory protein EIIA**^**Glc**^**and Nb form a super-complex with MelB**_**St**_**.***A*, the Nb effect of Nb733 on the binding of the native binding protein EIIA^Glc^ to MelB_St_ was determined by ITC measurement at 25 °C as described in Experimental procedures. An aliquot of 30 μM of free MelB_St_ or complexed with EIIA^Glc^ at a concentration of 60 μM (molar ratio of 1:2) in the buffer of 20 mM Tris–HCl, pH 7.5, 100 mM NaCl, 0.01% DDM, and 10% glycerol was placed in the sample cell. Solutions containing 180 μM Nb were prepared in a matching buffer and placed in the syringe. *B*, the EIIA^Glc^ effect on the Nb733 binding to MelB_St_. An aliquot of 50 μM of free MelB_St_ or complexed with Nb733 at a concentration of 75 μM (molar ratio of 1:1.5) in the buffer 20 mM Tris–HCl, pH 7.5, 100 mM NaCl, 0.01% DDM, and 10% glycerol was placed in the sample cell. Solutions containing EIIA^Glc^ (450 μM) were prepared in a matching buffer and placed in the syringe. *C*, Na^+^ binding to free WT MelB_St_ or complexed with EIIA^Glc^. An aliquot of 80 μM of MelB_St_ without or with EIIA^Glc^ at a concentration of 120 μM (molar ratio of 1:1.5) in the buffer of 20 mM Tris–HCl, pH 7.5, 50 mM choline chloride, 0.01% DDM, and 10% glycerol was placed in the sample cell. Solutions containing Na^+^ (5 mM) were prepared in a matching buffer and placed in the syringe. The thermogram was plotted as the baseline-corrected heat rate (μJ/s; *left* axis) *versus* time (*bottom* axis) for the titrant to free MelB_St_, MelB_St_/Nb733, or MelB_St_/EIIA^Glc^ complex (*black*) or to buffer (*red*) under an identical scale. Δ*H* (kJ/mol; filled *green* symbol) was plotted against the Nb/MelB_St_ molar ratio based on the *top*/*right* axes. DDM, n-dodecyl-β-D-maltopyranoside; ITC, isothermal titration calorimetry; MelB_St,_*Salmonella enterica* serovar Typhimurium melibiose permease.

EIIA^Glc^, which is the central regulator in the glucose-specific phosphoenolpyruvate/sugar phosphotransferase system in certain bacteria (23). Previous studies showed that EIIA^Glc^ binds readily to the apo (28) and Na^+^-bound MelB_St_ at a *K*_d_ of ∼3 μM (24). Surprisingly, EIIA^Glc^ also binds to the MelB_St_/Nb733 complex with a 2-fold reduced affinity (Fig. 6*A*; Table 3); similarly, the Nb733 binding affinity to MelB_St_ was also not affected significantly by the prebinding with EIIA^Glc^ (Fig. 6*B*). The supercomplex MelB_St_/Nb733/EIIA^Glc^ can be isolated from gel-filtration chromatography. EIIA^Glc^ protein does not absorb at 280 nm due to a lack of Trp and Tyr residues; however, EIIA^Glc^ shifted the MelB_St_ peak from 13.02 ml to 12.91 ml and further shifted the MelB_St_/Nb733 complex peak to 12.73 ml. The peak fractions analyzed by SDS-15%PAGE showed that MelB_St_, Nb733, and EIIA^Glc^ are present in all fraction of the supercomplex peak at 12.73 ml, providing strong evidence that both Nb733 and EIIA^Glc^ can cocurrently interact with MelB_St_ (Fig. S2, *A* and *B*).

Previously published results also showed that EIIA^Glc^ largely inhibits the sugar binding in MelB (24) and another sugar transport lactose permease LacY (25). To test if the EIIA^Glc^ binding behaves similarly to the Nb733, Na^+^ binding to MelB_St_/EIIA^Glc^ complex was also analyzed (Fig. 6*C*). Similarly, the MelB_St_/EIIA^Glc^ complex also retains the Na^+^ binding affinity at a *K*_d_ value of just 2-fold higher than that of the free MelB_St_ (Table 3).

## Discussion

Nbs have been shown to be useful for membrane protein structure determination by X-ray crystallography or cryo-EM single-particle analysis (3, 4, 5, 29, 30). Membrane transporters have intrinsically conformational flexibility necessary for their functions. Current advances in structural biology have confirmed that a solvent-access path on both sides of the membranes is alternatively formed and closed for substrate binding and translocation across the membranes (31, 32, 33, 34). The available knowledge also suggests that most membrane transporters have their favored conformations. As such, it is challenging to determine high-resolution structures in their transient, less populated states. In addition, the MFS transporters are a group of so-called hydrophobic membrane proteins with the majority mass embedded within the membranes. All those structural features increase the difficulties in high-resolution structure determination for the mechanistic studies of transport processes. Nbs usually bind to a tertiary epitope with high affinities and can select/stabilize conformations and reduce intrinsic conformation dynamics/motions (1, 3). Nbs have attracted increasing attention in protein research.

It is challenging to obtain Nbs to target a specific conformation. In this study, we tested if a specific selection condition can provide conformation-targeted Nbs. We applied 4 selection conditions and analyzed the data based on the Nb group, occurrence under each selection condition, and extensive characterizations of interactions with the targeted protein, both *in vivo* and *in vitro*. Interestingly, the group 1 members were isolated from all 4 conditions at only 5 occurrences (Table 1), and Nbs with identical amino acid sequences were obtained from different selection conditions, such as the Nb718 and Nb733. In contrast, the group 4 members with higher 99 occurrences were from 3 conditions including melibiose-containing and Li^+^ alone, but not Na^+^ alone conditions. This high occurrence implies that the group 4 members might bind to an epitope that is available at all or most conformational states of the transporter. This is also supported by their partial inhibition of the melibiose transport (Table 1; Fig. 3). The low occurrences from group 1 might suggest that this group member might bind MelB_St_ at certain transient high-energy states. Notably, the group 1 Nbs can be obtained from either condition, Na^+^ or Li^+^, alone or together with melibiose. The same group Nbs might bind to a similar epitope, which did not exclude the possibility that a similar epitope can be recognized by Nbs from more than one group. To establish a general method to analyze soluble protein and membrane protein interactions *in vivo*, a widely used soluble protein-protein interaction assay based on reconstituted CyaA activities by 2 hybrids (17, 18, 19, 20, 21) was set up. After testing the known dimeric proteins (ZIP or MelR), this simple test provided evidence of the direct interaction and confirmed a previously proposed interaction between MelR and a created ankyrin protein ANK-N5C-281 (27). This *in vivo* method also suggested that nearly all Nbs bind to the cytoplasmic side of MelB_St_, except for the Nb723:T18 fusion that did not form any colonies and had no readout (Fig. 3*A*). The conclusion was further confirmed by the [^3^H]melibiose active transport assay with intact cells when intracellularly co-expressing the soluble Nbs with the membrane protein MelB_St_ (Fig. 3*B*). Thus, the two-hybrid assay could provide valid information for intracellular Nb-membrane protein interactions. On the other hand, the results imply that the sidedness of MelB_St_ in proteoliposomes is predominantly inside-out orientated. This is consistent with the previous conclusion in MelB_Ec_, where MelB_Ec_ in proteoliposomes was reported as uniformly inside-out oriented as determined by chemical modification (35) and the recent study with single-molecule force spectroscopy of MelB_St_ (36).

The group 1 members (714, 725, and 733) are structurally stable and caused full inhibition of Na^+^-coupled melibiose active transport and the downhill melibiose transport activities, and hence were prioritized for further analysis. All 3 Nbs are highly conserved with only 2 differences in the CDR-2/3 and one difference in the scaffold region; all bind MelB_St_ with a *K*_d_ value in a range of 0.52 to 1.61 μM and possess similar effects on MelB_St_ functions. The Nbs binding was largely inhibited by melibiose, and the melibiose binding to MelB_St_ was also inhibited by the Nbs, suggesting that this group of Nbs functions as negative allosteric modulators; thus, the anticooperativity implies that the melibiose-favored conformation conflicts with the Nb-favored conformation. It is likely that the state of the Nb-bound MelB_St_ largely differs from the available sugar-bound outward-facing structures solved by X-ray crystallography.

The Nb733-bound MelB_St_ retains binding to the coupling cation Na^+^ and the physiological regulator EIIA^Glc^. Interestingly, the observed non-cooperative binding of Nbs with Na^+^ and anti-cooperativity with melibiose are similar to what was observed for EIIA^Glc^ (24, 28) (Table 3). The data further confirmed that MelB_St_ trapped by this group of Nbs retains a physiological conformation, likely a Na^+^-bound sugar conformation.

This study established a general *in vivo* assay to test *in vivo* protein-protein interactions and the binding sidedness to membrane proteins in *E. coli* and also identified a few potential useful Nbs for future structural determination of MelB_St_. It is noteworthy that the critical coupling cations have not been resolved for any of the MFS transporters. The MelB Nbs could be a valuable tool to facilitate the structural determination of a Na^+^-bound MelB_St_ and deepen our understanding of the ion-coupled transported mechanisms.

## Experimental procedures

### Reagents

[1-^3^H]Melibiose (5.32 Ci/mmol) was custom synthesized by PerkinElmer, and unlabeled melibiose was purchased from Acros Organics (Thermo Fisher Scientific). MacConkey agar media (lactose-free) was purchased from Difco. Detergents UDM, DDM, and octyl-β-D-glucoside were purchased from Anatrace *E. coli* lipids (Extract Polar, 100600) were purchased from Avanti Polar Lipids, Inc. Oligodeoxynucleotides were synthesized by Integrated DNA Technologies. All other materials were reagent grade and obtained from commercial sources.

### Strains

The genotype and source of *E. coli* strains used or created in this study are described in Supplementary Notes and listed in Tables S1 and S2 unless otherwise described specifically. Briefly, the commercial *E. coli* Stellar or XL1 Blue were used for plasmid construction. The *E. coli* DB3.1 strain was used to construct the *ccdB*-containing universal FX cloning vectors (37). *E. coli* DW2 *(melB-lacY*^*-*^) (38) was used for functional studies, and 3 *cyaA*^-^ strains (DH5*α*, *DW2*, and *T7 Express*) were generated for the *in vivo* protein-protein interaction assay. *E. coli* BW25113 strain and plasmids pKD46, pKD4, and pCP20 (7) were used for gene deletion.

### Plasmids construction

The plasmids used or created in this study are listed in Supplementary Notes and Table S1. The primers used for this study are listed in Table S2.

### MelB_St_ protein expression and purification

Cell growth for the large-scale production of WT MelB_St_ was carried out using the pK95 ΔAH/MelB_St_/CHis_10_ from *E. coli* DW2 cells (*melA*^*+*^*melB*, *lacZY*) as described (10, 39). Briefly, MelB_St_ purification from membranes by cobalt-affinity chromatography (Talon Superflow Metal Affinity Resin, Takara) after extraction by 1.5% UDM or 2% DDM. MelB_St_ protein was eluted with 250 mM imidazole in a buffer containing 50 mM NaPi, pH 7.5, 200 mM NaCl, 0.035% UDM or 0.01% DDM, and 10% glycerol, and dialyzed to change the buffer conditions accordingly.

### MelB_St_ reconstitution

The MelB_St_ reconstitution into proteoliposomes was carried out by dilution using *E. coli* Extract Polar (Avanti) as described (13). Briefly, 46.25 mg of the lipids dissolved in 1.2% octyl-β-D-glucoside was mixed with 9.25 mg of the MelB_St_ in UDM at a ratio of 1:5 (mg:mg). After a 30 min incubation at room temperature, the mixture was subjected to a 74-fold dilution with a buffer of 20 mM NaP_i_, pH 7.5, and 150 mM NaCl, and further incubated for another 30 min with stirring. Proteoliposomes were harvested by ultracentrifugation at 28,000 rpm using Beckman rotor 45 Ti at 4 °C overnight. They were further washed by resuspending the pellets in 20 ml of 20 mM NaP_i_, pH 7.2, 150 mM NaCl buffer, followed by 3 cycles of freeze-thaw-sonication and collected by ultracentrifugation at 47,000 rpm on a Beckman rotor 70 Ti at 4 °C for 2 h. The pellets were re-suspended in the same buffer and subjected to another 3 cycles of freeze-thaw-sonication. The sonication was carried out in an ice-cold bath sonicator (Branson 2510) for 5 s 3 times. The samples were washed once with 24 ml of the same buffer and concentrated to a protein concentration of 8.5 mg/ml by ultracentrifugation, which yielded about 8.7 mg of MelB_St_ proteoliposomes.

### Nbs generation and specific selection

The MelB_St_ proteoliposomes in 20 ml of 20 mM NaPi, pH 7.2, 150 mM NaCl buffer were used to raise Nbs as described (1). In brief, one llama (*Lama glama*) received 6 weekly injections of 100 μg of WT MelB_St_-containing proteoliposomes. The Nb-encoding ORFs were amplified from total lymphocyte RNA and subcloned into the phage display/expression vector pMESy4.

Four defined conditions were used to select specific Nbs by phage display. The 4 buffers are (1) 20 mM KPi, pH 7.4 and 150 mM NaCl (2), 20 mM KPi, pH 7.4, 150 mM NaCl, and 10 mM melibiose (3), 20 mM KPi, pH 7.4 and 150 mM LiCl, and (4) 20 mM KPi, pH 7.4, 150 mM LiCl, and 10 mM melibiose, The buffer for coating MelB_St_ on a solid phase and for the Nbs selections is identical. A total of 344 individual colonies were randomly picked, and the Nbs were produced as soluble His- and Capture Select C-tagged proteins (MW 12–15 kDa) in the periplasm of *E. coli*. DNA sequencing analysis resulted in 6 groups (Table 1)

### A bacterial two-hybrid assay

A bacterial two-hybrid assay was established, which is based on reconstituting CyaA activities from the T18/T25 fragments of *B. pertussis* CyaA toxin as described (18, 22). The *cyaA* gene product in *E. coli* solely responds to cAMP synthesis. Three *E. coli* strains, including DH5α, DW2 (*melB*^*-*^), and T7 Express, were subjected to *cyaA* gene deletion by one-step gene deletion method (7, 40, 41). A 2166 bp chromosomal DNA fragment coding for 722 amino acid residues (Leu^51^-Ser^772^) of CyaA of each strain was removed, and the resulting resistance cassette was also eliminated (Table S1). The deletion was confirmed by DNA sequencing analysis of the PCR product amplified from the chromosomal DNA of each strain, which revealed a short fragment (∼300 bp) instead of the 2166 bp fragment.

Expression vectors carrying T18 or T25 fragments were engineered to suit the FX Cloning method (27, 37). Resulting universal vectors carrying T18 or T25 fragments were used to generate 2 fusions with two target genes at the N-terminus of T18 or the C-terminus of T25 for FX Cloning, or at another polarity. Two known dimer proteins, including ZIP and the melibiose operon transcription activator (MelR), were selected for the positive control (Table S1). A previously engineered transcription inhibitor, which was based on ankyrin proteins (ANK-N5C) by a direct evolution method (27), with the MelR and the Nbs with MelB_St_ were subjected to test the potential intracellular interactions (Table S1).

The DH5α competent cells without the gene coding for the endogenous *cyaA*^-^ strain were selected for the tests due to suitable fermentation signals. The cells were transformed by 2 compatible plasmids with different replication origins (pACYC and pCS19) (42) containing the 2 hybrids and plated onto the lactose-free MacConkey agar plate containing 30 mM maltose as the sole carbohydrate source, 100 mg/L ampicillin, 25 mg/L chloramphenicol, and 0.2 mM IPTG, and the plates were incubated in 30 °C for 7 days before photography. Red colonies grown on the MacConkey agar indicate positive fermentation due to the cAMP production derived from the interaction of 2 hybrids. Yellow colonies denote no fermentation and no cAMP production.

### [1-^3^H]Melibiose transport assay and Western blot

*E. coli* DW2 cells (*mel*A^+^, *melB*^*-*^, and *lacZ*^*-*^*Y*^*-*^) (39) transformed with the 2 compatible plasmids (pACYC and pCS19) with no or with MelB_St_ and Nbs, respectively, were grown in LB broth with 100 mg/L ampicillin and 25 mg/L chloramphenicol in a 37 °C shaker overnight. The overnight cultures were diluted by 5% to fresh LB broth with 0.5% glycerol, 100 mg/L ampicillin, 25 mg/L chloramphenicol, and 0.2 mM IPTG shaken at 30 °C for 5 h. The transport assay was performed at 20 mM Na^+^ and 0.4 mM [^3^H]melibiose (specific activity of 10 mCi/mmol) as described (26, 43). The cells were washed with 50 ml of 100 mM KP_i_, pH 7.5 3 times, followed by washing with the assay buffer (100 mM KP_i_, pH 7.5, 10 mM MgSO_4_). The cell pellets were resuspended with the assay buffer and adjusted to *A*_420_ = 10 (∼0.7 mg proteins/ml). The transport time courses were carried out by separating the cells from solutions at zero, 5 s, 10 s, 30 s, 1 m, 2 m, 5 m, 10 m, and 30 m by dilution and fast filtration. The filters were subjected to radioactivity measurements using a liquid scintillation counter.

MelB_St_ membrane expression was analyzed by Western blot. Cultured cells were washed with 50 mM NaPi (pH 7.5), resuspended with the same buffer, and broken by sonication. The unbroken cells or debris were removed by centrifugation, and the supernatant were subjected to ultracentrifugation at 62,000 rpm for 30 min in a Beckman rotor Ti 70. The membrane pellets were resuspended with 50 mM NaPi, pH 7.5, 200 mM NaCl, and 10% glycerol. After protein concentration determination, 50 μg of total membranes were loaded onto SDS-15%PAGE. Western blot analysis using anti-His antibody was carried out. MelB_St_ protein was detected using the SuperSignal West Pico Chemiluminescent Substrate (Thermo Fisher Scientific) by the ChemiDoc MP Imaging System (Bio-Rad).

### Melibiose fermentation on MacConkey agar plates

*E. coli* DW2 cells (Δ*melBΔlacYZ*) were transformed with 2 compatible plasmids with different replication origins (pACYC/MelB_St_ and pCS19/Nbs) and plated onto the lactose-free MacConkey agar plate containing 30 mM melibiose as the sole carbohydrate source, 100 mg/L ampicillin and plated on MacConkey agar plates containing 30 mM melibiose, 100 mg/L ampicillin, 25 mg/L chloramphenicol, and 0.2 mM IPTG. The plates were incubated at 37 °C for 18 h before photography. Magenta colonies, a normal melibiose fermentation; pink colonies, reduced fermentation; and yellow colonies, no melibiose fermentation.

### Protein concentration assay

The Micro BCA Protein Assay (Pierce Biotechnology, Inc) was used to determine the protein concentration assay.

### Nbs expression and purification

Cytoplasmic expression of Nbs was carried out using the expression plasmids p7xC3H (37) in the *E. coli* ArcticExpress (DE3) strain (Table S1). The cells were grown in LB broth with 0.5% glucose, and 50 mg/L kanamycin at 37 °C. Overnight cultures were inoculated at a 1:100 ratio into LB broth containing 0.5% glucose and 50 mg/L kanamycin and shaken at 37 °C; 0.5 mM IPTG was added when *A*_600_ equaled 0.8, and cells were then shaken at 12 °C overnight. Cells were washed once with 50 mM NaPi, pH 7.5, resuspended in 50 mM NaPi, pH 7.5, and 1 mM PMSF, and broken by passage through Emulsiflex twice. After ultracentrifugation at 70,409*g* for 30 min using Beckman rotor, type 45 Ti at 4 °C, the supernatants containing soluble Nbs were collected and subjected to cobalt-NTA (Talon resin)-affinity chromatography. The column was preequilibrated with 50 mM NaPi, pH 7.5, 500 mM NaCl, 10% glycerol, and 5 mM imidazole. After washing with the same buffer containing 45 mM imidazole, elution was performed using the same buffer containing 250 mM imidazole. Buffer exchange was performed to match the MelB_St_ solution by overnight dialysis. The protein concentration of Nbs was measured by the Micro BCA protein assay (Pierce Biotechnology, Inc).

### EIIA^Glc^ expression and purification

The overexpression of unphosphorylated EIIA^Glc^ was performed in the *E. coli* T7 Express strain (New England Biolabs) as described (24).

### Isothermal titration calorimetry

All ITC ligand-binding assays were performed with the TA Instruments (Nano ITC device) as described (12). In a typical experiment, the titrand (MelB_St_) placed in the ITC sample cell was titrated with the specified titrant (placed in the syringe) by an incremental injection of 1.6- or 2 μL aliquots at an interval of 300 s at a constant stirring rate of 250 rpm (nano ITC). All samples were degassed using a TA Instruments Degassing Station (model 6326) for 15 min prior to the titration. Heat changes were collected at 25 °C, and data processing was performed with the NanoAnalyze (version 3.7.5 software) provided with the instrument. The normalized heat changes were subtracted from the heat of dilution elicited by the last few injections, where no further binding occurred, and the corrected heat changes were plotted against the molar ratio of titrant *versus* titrand. The values for the binding association constant (*K*_a_) and enthalpy change (Δ*H*) were obtained by fitting the data using the one-site independent-binding model included in the NanoAnalyze software (version 3.7.5). The binding free energy (Δ*G*) = -*RT* ln *K*_a_, where *R* is the gas constant (8.315 J/mol·K), and *T* is the absolute temperature; Δ*G* = Δ*H* - *T*Δ*S*; the entropy change (-*T*Δ*S*) = Δ*G –* Δ*H*; dissociation constant (*K*_d_) = 1/*K*_a_.

## Data availability

Source data are available from the corresponding author.

## Supporting information

This article contains supporting information (7, 17, 22, 24, 26, 27, 37, 38, 40, 41, 42).

## Conflict of interest

The authors declare that they have no conflicts of interest with the contents of this article.
