# Supplementary material for: In vivo and in vitro characterizations of melibiose permease (MelB) conformation-dependent nanobodies reveal sugar-binding mechanisms
Source: J Biol Chem. 2023 Jun 26;299(8):104967. doi: 10.1016/j.jbc.2023.104967 (PMC10374971; doi:10.1016/j.jbc.2023.104967)
Supplement: Supporting Information [file mmc1.pdf]

In vivo and *in vitro* characterizations of melibiose permease (MelB) conformation-dependent nanobodies reveal sugar-binding mechanisms

Satoshi Katsube[1], Katleen Willibal[2,3], Sangama Vemulapally[1], Parameswaran Hariharan[1], Elena Tikhonova[1], Els Pardon[2, 3], H. Ronald Kaback[4#], Jan Steyaert[2, 3], and Lan Guan[1§]

[1]Department of Cell Physiology and Molecular Biophysics, Center for Membrane Protein Research, School of Medicine, Texas Tech University Health Sciences Center, Lubbock, TX; [2]VIB Center for Structural Biology Research, VIB, 1050 Brussel, Belgium; [3]Structural Biology Brussels, Vrije Universiteit Brussel, Pleinlaan 2, 1050 Brussel, Belgium; [4]Department of Physiology and Department of Microbiology, Immunology, and Molecular Genetics, Molecular Biology Institute, University of California, Los Angeles, Los Angeles, CA.

[#]Deceased

[§]To whom correspondence should be addressed:

Lan Guan, **E-mail**: Lan.Guan@ttuhsc.edu

Supplemental Tables and Notes

sTable 1. *E. coli* strains and plasmids used in this study

Strains and vectors	Description	Reference
**Stains**		
T7 Express	*fhuA2 lacZ::T7 gene1 [lon] ompT gal sulA11 R(mcr-73::miniTn10*--Tet^S*)2 [dcm] R(zgb-210::Tn10*--Tet^S*) endA1 Δ(mcrC-mrr)114::IS10*	NEB
DW2	*melA*$^+$ Δ*melB* Δ*lacZY*	(38)
DH5α	supE44 ΔlacU169 (Φ80 lacZΔM15) hsdR17 relA1 gyrA96 thi-1 recA1	
XL1 Blue	*recA1 endA1 gyrA96 thi-1 hsdR17 supE44 relA1 lac [F' proAB lacIqZΔM15 Tn10 (Tetr)]*	Agilent Technologies
Stellar^{TM}	*F', endA1, supE44, thi-1, recA1, relA1, gyrA96, phoA, Φ80d lacZΔ M15, Δ(lacZYA-argF) U169, Δ(mrr-hsdRMS-mcrBC), ΔmcrA, λ-*	Clontech
ArcticExpress (DE3)	F$^-$ *ompT hsdS*(rB $^-$ mB $^-$) *dcm*$^+$ Tetr gal λ(DE3) *endA* Hte [cpn10 cpn60 Gentr]	Agilent Technologies
DB 3.1	*gyrA*462 *endA*1 Δ(sr1-recA) *mcrB mrr hsd*S20 glnV44 (=*supE*44) ara14 *galK*2 *lacY*1 *proA*2 *rpsL*20 *xyl*5 *leuB*6 *mtl*1	Invitrogen
BW25113	*rrnB3 DElacZ4787 hsdR514 DE(araBAD)567 DE(rhaBAD)568 rph-1*	(7)
DW2 *cyaA*$^-$	Δ*cyaA*	This study
DH5α *cyaA*$^-$	Δ*cyaA*	This study
E. coli T7 Express *cyaA*$^-$	Δ*cyaA*	This study
**Plasmids**		
pKD46	Red recombinase expression plasmid contains Amp resistance, temperature-sensitive replication	(7)
pKD4	Kanamycin-resistant cassette (FRT-Km-FRT cassette)	(7)
pCP20	Cam-resistant, temperature-sensitive replication, thermal induction of FLP synthesis	(7)
pCS19	pQE60 derivative inserted with gene *lacI*^{q}; amp^r	(42)
pACYC	pACYC/FX-derived vector for control; no *ccdB* gene	(27)
p7xC3H/FX	Expression vector with two SapI sites and *ccdB* gene for FX cloning; km^r	(37)
pCS19/FX	Expression vector with two SapI sites and *ccdB* gene for FX cloning; amp^r	(27)
pACYC/FX	Expression vector with two SapI sites and *ccdB* gene for FX cloning; cam^r; compatible with pCS19/FX-derived plasmids	(27)
pACYC/MelB$_{St}$	Expression vector for MelB$_{St}$ derived from pACYC/FX; cam^r	(27)
p7xNH3/IIA^{Glc}	Expression vector for EIIA^{Glc} derived from p7xNH3/FX; km^r	(24)
pK95 ΔAH/MelB$_{St}$/ CHis10	MelB$_{St}$ with a C-terminal His10 tag; amp^r	(26)
pCS19/FX2	A modified pCS19/FX with introduction of two restriction sites (BamH I and Spe I) following the 3' Sap I FX Cloning site.	This study
pCS19/X:T18/FX	pCS19/FX2-derived vector for expressing a target protein "X" with a C-terminal fusion with T18 fragment; two SapI sites and *ccdB* gene for FX cloning; amp^r	This study
pCS19/T18	pCS19/X:T18/FX-derived vector for expressing T18 fragment; no *ccdB* gene.	This study
pCS19/ZIP:T18	Expression vector for ZIP:T18 fusion derived from pCS19/X:T18/FX; amp^r	This study
pCS19/MelR:T18	Expression vector for MelR:T18 fusion derived from pCS19/X:T18/FX; amp^r	This study
pACYC/T25:X/FX	pACYC/FX-derived vector for expressing a target protein "X" with N-terminal fusion with T25 fragment; two SapI sites and *ccdB* gene for FX cloning; a 6xHis Tag is fin-frame with T25; cam^r	This study
pACYC/FX2	A modified pACYC/FX with introduction of Nco I site containing the first ATG codon to 5' of the 1st Sap I site and introducing a Spe I site following 2nd Sap I site.	This study
pACYC/T25	pACYC/T25:X/FX-derived vector for expressing T25 fragment; no *ccdB* gene	This study

pACYC/T25:ZIP	Expression vector for T25:ZIP fusion derived from pACYC/T25:X/FX; cam^r	This study
pACYC/T25:MelR	Expression vector for T25:MelR fusion derived from pACYC/T25:X/FX; cam^r.	This study
pACYC/T25:$MelB_{St}$	Expression vector for T25: $MelB_{St}$ fusion derived from pACYC/T25:X/FX; cam^r	This study
pACYC/T25:ANK-N5C-281	Expression vector for T25:ANK-N5C-281 fusion derived from pACYC/T25:X/FX; cam^r.	This study
pACYC/T25:ANK-N5C-62	Expression vector for T25:ANK-N5C-62 fusion derived from pACYC/T25:X/FX; cam^r.	This study
pACYC/T25:ANK-N5C-64	Expression vector for T25:ANK-N5C-64 fusion derived from pACYC/T25:X/FX; cam^r.	This study
pACYC/T25:ANK-N5C-88	Expression vector for T25:ANK-N5C-88 fusion derived from pACYC/T25:X/FX; cam^r.	This study
pACYC/T25:ANK-N5C-317	Expression vector for T25:ANK-N5C-317 fusion derived from pACYC/T25:X/FX; cam^r.	This study
pACYC/T25:ANK-N5C-429	Expression vector for T25:ANK-N5C-429 fusion derived from pACYC/T25:X/FX; cam^r.	This study
pCS19/ANK-N5C-281:T18	Expression vector for ANK-N5C-281:T18 fusion derived from pCS19/X:T18/FX; amp^r	This study
pCS19/ANK-N5C-62:T18	Expression vector for ANK-N5C-62:T18 fusion derived from pCS19/X:T18/FX; amp^r	This study
pCS19/ANK-N5C-64:T18	Expression vector for ANK-N5C-64:T18 fusion derived from pCS19/X:T18/FX; amp^r	This study
pCS19/ANK-N5C-88:T18	Expression vector for ANK-N5C-88:T18 fusion derived from pCS19/X:T18/FX; amp^r	This study
pCS19/ANK-N5C-317:T18	Expression vector for ANK-N5C-317:T18 fusion derived from pCS19/X:T18/FX; amp^r	This study
pCS19/ANK-N5C-429:T18	Expression vector for ANK-N5C-429:T18 fusion derived from pCS19/X:T18/FX; amp^r	This study
pCS19/Nb712:T18	Expression vector for Nb712:T18 fusion derived from pCS19/X:T18/FX; amp^r	This study
pCS19/Nb714:T18	Expression vector for Nb714:T18 fusion derived from pCS19/X:T18/FX; amp^r	This study
pCS19/Nb715:T18	Expression vector for Nb715:T18 fusion derived from pCS19/X:T18/FX; amp^r	This study
pCS19/Nb721:T18	Expression vector for Nb721:T18 fusion derived from pCS19/X:T18/FX; amp^r	This study
pCS19/Nb723:T18	Expression vector for Nb723:T18 fusion derived from pCS19/X:T18/FX; amp^r	This study
pCS19/Nb725:T18	Expression vector for Nb725:T18 fusion derived from pCS19/X:T18/FX; amp^r	This study
pCS19/Nb728:T18	Expression vector for Nb728:T18 fusion derived from pCS19/X:T18/FX; amp^r	This study
pCS19/Nb732:T18	Expression vector for Nb732:T18 fusion derived from pCS19/X:T18/FX; amp^r	This study
pCS19/Nb733:T18	Expression vector for Nb733:T18 fusion derived from pCS19/X:T18/FX; amp^r	This study
pCS19/Nb738:T18	Expression vector for Nb738:T18 fusion derived from pCS19/X:T18/FX; amp^r	This study
pCS19/Nb712	Expression vector for Nb712 derived from pCS19/FX; amp^r	This study
pCS19/Nb714	Expression vector for Nb714 derived from pCS19/FX; amp^r	This study
pCS19/Nb715	Expression vector for Nb715 derived from pCS19/FX; amp^r	This study
pCS19/Nb721	Expression vector for Nb721 derived from pCS19/FX; amp^r	This study
pCS19/Nb723	Expression vector for Nb723 derived from pCS19/FX; amp^r	This study
pCS19/Nb725	Expression vector for Nb725 derived from pCS19/FX; amp^r	This study
pCS19/Nb728	Expression vector for Nb728 derived from pCS19/FX; amp^r	This study
pCS19/Nb732	Expression vector for Nb732 derived from pCS19/FX; amp^r	This study
pCS19/Nb733	Expression vector for Nb733 derived from pCS19/FX; amp^r	This study
pCS19/Nb738	Expression vector for Nb738 derived from pCS19/FX; amp^r	This study

p7xC3H/Nb714	Expression vector for Nb714 derived from p7xC3H/FX; km^r	This study
p7xC3H/Nb725	Expression vector for Nb725 derived from p7xC3H/FX; km^r	This study
p7xC3H/Nb733	Expression vector for Nb733 derived from p7xC3H/FX; km^r	This study

sTable 2 Primers uses and created in this study

Applications	Labels	Oligonucleotides
E. coli cyaA deletion	cyaA_dele	P1-s: 5′-TTGCCTTTACACGCCTGATGAAACTCAACGCCACTACCT GAACGAGCTGTGTAGGCTGGAGCTGCTTCG -3′
		P2-as: 5′-CTTCACAATCTGATAGAACTGCGGCAGGTTGAAGTTGAT GAAGCTTCATATGAATATCCTCCTTAG -3′
Chromosomal DNA sequencing		T1-s: 5'–ATAAATCAATTGCGTGTG–3'
		T2-as: 5'–TGCGGAACGGAATCACC–3'
		k1-s: 5'–CAGTCATAGCCGAATAGCCT –3'
		k2-as: 5'–CGGTGCCCTGAATGAACTGC –3'
ZIP construct	ZIP1	P1-s: 5'-CGTATGAAACAGTTGGAAGATAAGGTAGAGGAACTGC TGAGCAAGAACTATCACTTGGAG-3'
	ZIP2	P2-as: 5'-GCGTTCACCGACCAGTTTCTTTAAACGGGCTACCTCG TTCTCCAAGTGATAGTTCTTGC-3'
pACYC/FX2 construction	NcoI insertion	Fwd: 5′-CTTCGAGTTGCAGAAACAGCCATGGGCTCTTCGAGTTGCAG-3′
		Rev: 5′- CTGCAACTCGAAGAGCCCATGGCTGTTTCTGCAACTCGAAG-3′
	SpeI insertion	Fwd: 5′-GTTATCTGCACGAAGAGCACTAGTCGTCGTTTTACAACGTC GTG-3′
		Rev: 5′-CACGACGTTGTAAAACGACGACTAGTGCTCTTCGTGCAGAT AAC-3′
pCS19/FX2 construction	BamH1-SpeI insertion	Fwd: 5′-ATCTGCATGAAGAGCTGAGGATCCACTAGTGTTGATAGATCC AGTAATG-3′
		Rev: 5′- CATTACTGGATCTATCAACACTAGTGGATCCTCAGCTCTTCA TGCAGAT-3′
p7xC3H/Nbxxx	Nb-FX	Fwd: 5′-ATATATGCTCTTCTAGTCAGGTGCAGCTG-3′
		Rev: 5′-TATATAGCTCTTCATGCACTGGAGACGGTGACCTG-3′
pCS19/Nbxxx:T18 (This adds C-terminal 'A' and allow the C-terminal fusion)	MelB_Nb	Fwd: 5′-ATATATGCTCTTCTAGTCAGGTGCAGCTG-3′
		Rev: 5′-TATATAGCTCTTCATGCACTGGAGACGGTGACCTG-3′
pCS19:Nbxxx-CTH (This adds C-terminal 'HHHHHH and truncated by a stop codon)	MelB_FX	Fwd: 5′-ATATATGCTCTTCTAGTATGCAGGTGCAGCTG-3′
		Rev: 5′-TATATAGCTCTTCATGCTTAGTGGTGATGATGGTGGTGACT GGAGACGGTGACCTG-3′
pACYC/T25:MelB$_{St}$	MelB_FX	Fwd: 5′-ATATATGCTCTTCTAGTAGCATTTCAATGACTACAAAAC-3′
		Rev: 5′-TATATAGCTCTTCATGCTCAATGGTGATGGTGATGGTGA TG-3′
pCS19/ANK-N5C-xxx:T18 pACYC/T25:ANK-N5C-xxx	ANK-N5C-FX	Fwd: 5′-TGGCCGCTCTTCTAGTATGGATATCGGTAAGAAGCTTCTCGA AGCC-3′
		Rev: 5′-GGTGGCCGCTCTTCATGCTTAGTGATGGTGATGGTGATGAG ATCTAGC-3′
pACYC/T25:ZIP pCS19/ZIP:T18	Zip-FX	Fwd: 5′-ATATATGCTCTTCTAGTCGTATGAAACAGTTGGAAG-3′
		Rev: 5′-GGTGGCCGCTCTTCCTGCGCGTTCACCGACCAGTTTC-3′
pACYC/T25:MelR pCS19/MelR:T18	MelR-FX	Fwd: 5′- ATATATGCTCTTCTAGTAATACAGATACGTTTATGTGCAGC-3′
		Rev: 5′- GGTGGCCGCTCTTCCTGCGCCGGGAAACGTCTGGCGGCGC TG-3′

Supplementary Notes

Construction of chromosomal *cyaA gene* deletion mutant strain of *E. coli* DH5*α*, DW2, and T7 Express. Wanne's gene-deletion method (7, 40-41) was used for deleting the *cyaA* gene, which is solely responsible for cAMP production. Plasmids pKD46, pKD4 and pCP20 (available from Dr. Barry L. Wanner). Oligonucleotide primers P1-s and P2-as, which contain 44-45 nucleotide extensions homologous to sequences flanking the *cya*A gene on the *E. coli* chromosome, were used for amplifying a recombination PCR fragment containing kanamycin resistance gene (Km) flanked by two flippase recognition target (FRT) sites on the plasmid pKD4. Primers T1-s and T2-as were used for amplifying *cyaA* gene from 43 to 2494; the primers k1 and k2 are the same as the original report (7). After the homologous recombination catalyzed by a λ Red recombinase encoded by the plasmid pKD46 between the recombination PCR fragment and the *E. coli* chromosome, the *cya*A gene is exchanged for the kanamycin resistance gene, which was then eliminated by site-specific recombination between the two FRT sites catalyzed by an FLP recombinase encoded by pCP20. Growing at 42 °C eliminated the plasmids pKD46 and then the pCP20, which are temperature sensitive. The kanamycin sensitivity of all three strains were tested by growing on Km-containing plates. Finally, at the locus of the *cya*A gene, a single FRT site remains, which replaces the *cyaA* gene between positions 43-2494. The PCR-amplified region by primers T1 and T2 within the *cyaA* gene exhibited about the 365 bp fragment, instead of 2,547-bp fragment, due to the deletion.

FX Cloning-derived plasmid design and construction. One of the advantages of FX Cloning system is the cross-compatibility and ease to perform subcloning to various FX Cloning vectors. Typically, a PCR fragment amplified by a pair of gene-specific and FX-compatible primers can be subcloned in any FX compatible vector of choice to enable parallel application in functional

(transport, fermentation, and two-hybrid assays) and protein expression studies. For setting up a two-hybrid system, two-hybrid assay vectors were constructed to be compatible with the universal FX Cloning strategy. Two compatible expression plasmids pCS19/X:T18/FX and pACYC/T25:X/FX were constructed to allow a target protein "X" with a C-terminal fusion with T18 fragment (the C-terminal 225-339 region) or with an N-terminal fusion with T25 fragment (the N-terminal 1-224 position) of the catalytic domain of the adenylate cyclase of *Bordetella pertussis*, respectively (17, 22). All contain a *ccdB* cassette flanked by two Sap I sites used for directional cloning. T18 and T25 DNA fragments were obtained by Gene Synthesis (ITD). The "X" was placed with a *ccdB* gene by SapI digestion sites and F Cloning (37).

(1) The universal vector for T18 fusion pCS19/X:T18/FX. The pCS19/FX vector is modified by adding the BamH I and Spe I sites immediately following 2nd Sap I cloning site at the 3'side, named as pCS19/FX2. Based on this vector, the CyaA-T18 fragment was inserted between BamH I and Spe I to generate pCS19/X:T18/FX. Any N-terminal fusion of candidate genes (X) with the T18 fragment (pCS19/X:T18/FX) can be obtained by FX Cloning methods based on specific FX cloning primers (see sTable 2). The two fusion partner proteins (N-terminal candidate protein and T18) are linked by a 12-residue-long fragment of sequence ARRAPAPAPAGT.

(2) The universal vector for T25 fusion pACYC/T25:X/FX. The original pACYC/FX Sap I sites are removed by introducing a Nco I and a Spe I site in the flanking region of Sap I cloning segment, designated as pACYC/FX2. Digestion of pACYC/FX2 with Nco I and Spe I removes the FX cloning sites and the *ccdB* cassette. Insertion of the N-terminal CyaA-T25 fragment followed by two newly introduced FX Sap I cloning sites enables the C-Terminal fusion and restores FX cloning capability. The *ccdB* cassette is re-inserted

between the two Sap I sites, named pACYC/T25:X/FX. The C-terminal fusion of any candidate genes (X) with the N-terminal T25 fragment in the vector can be generated by FX Cloning method using specific FX cloning primers (see sTable 2). The two fusion partner proteins (N-terminal T25 and candidate protein) are linked by a 12-residue-long fragment of sequence PAPAPAGTGSSS.

ZIP fusion constructs. A 35-amino acid segment of the *leucine zipper* (ZIP)-encoding fragment was constructed using two overlapping oligonucleotides (ZIP1 and ZIP2) by PCR, amplified by using a pair of FX cloning primers ZIP-FX (**sTable 2**), and cloning into both universal hybrid vectors, yielding two fusions pCS19/ZIP:T18 and pACYC/T25:ZIP.

MelR constructs. MelR forms a dimer to bind the *mel* operon. Thus, the plasmid pCS19/MelR (27) was cloned into both universal hybrid vectors by using a pair of FX cloning MelR-FX primers (**sTable 2**), yielding two fusions pCS19/MelR:T18 and pACYC/T25:MelR.

$MelB_{St}$ fusion constructs. The $MelB_{St}$ from pACYC/$MelB_{St}$ (27) was cloned into the universal hybrid vector pACYC/T25:X/FX to generate pACYC/T25:$MelB_{St}$ by MelB_FX primers.

Nb fusion constructs. All Nbs share identical N- and C-terminal sequences, using a single-set primers MelB_Nb (**sTable 2**), all Nb:T18 fusion fragments were cloned to the universal hybrid pCS19/X:T18/FX vector to generate pCS19/Nbxxx:T18/FX.

ANK-N5C constructs. The designed 5 ankyrin proteins (including ANK-N5C-281, -64, -88, -317 and -455) were described previously (27). All ANK-N5C share identical N- and C-terminal sequences, so using a single set of primers ANK-N5C-FX (**sTable 2**), all ANK-N5C fusions with T18 or T25 were constructed to the universal hybrid vectors pCS19/X:T18/FX and pACYC/T25:X/FX, respectively, to generate pCS19/ANK-N5C-xxx:T18/FX and pACYC/T25:ANK-N5C-xxx, where xxx indicates the identity of NAK-N5C proteins.

sFig. 1

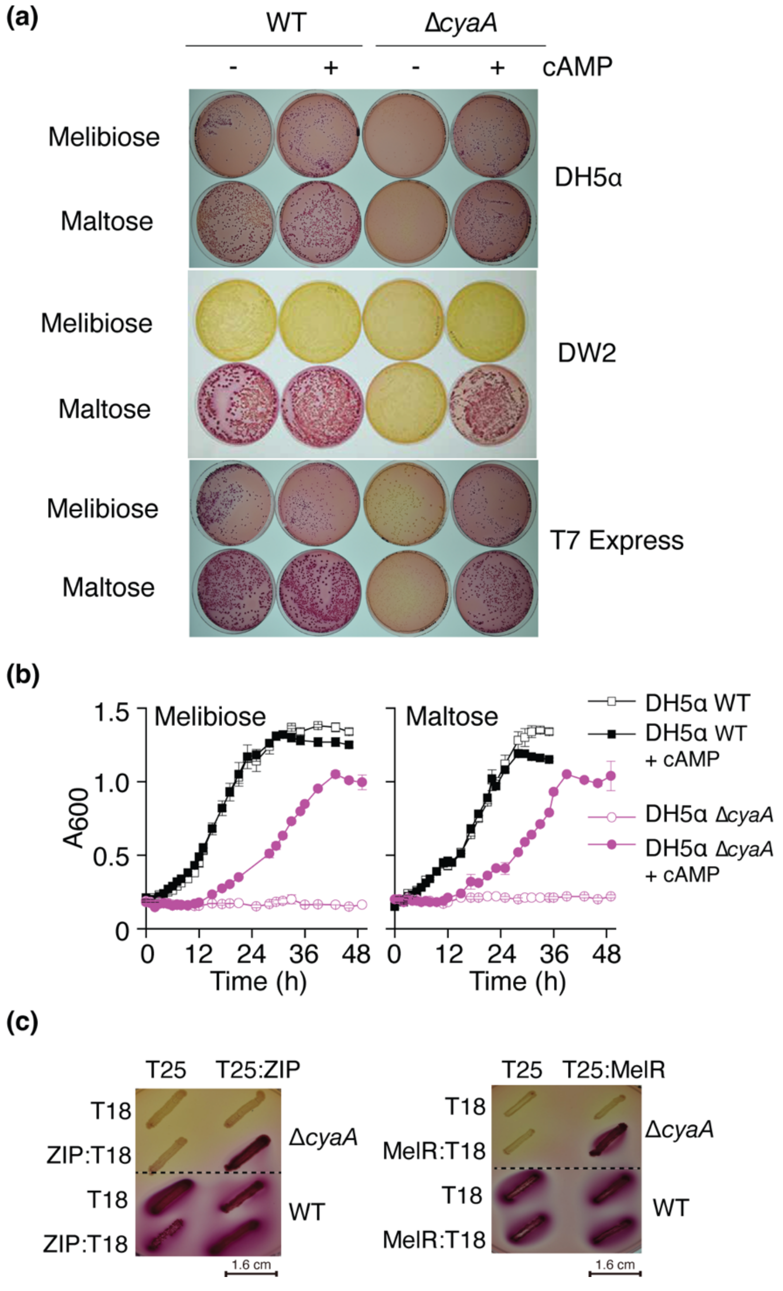

sFig. 1. Verification of the *E. coli ΔcyaA* strain. **(a)** Sugar fermentation. The *cyaA* gene deletion was applied to three *E. coli* strains DH5α, DW2, and T7 Express. The phenotype of the CyaA deletion was examined by the cAMP-dependent melibiose or maltose fermentation on MacConkey agar plates containing melibiose or maltose as the sole carbohydrate source. cAMP at a concentration of 0.5 mM was supplemented as a control. **(b)** Cell growth on M9 media. Cells were collected from overnight cultures in LB media, washed with M9 media, and re-inoculated into M9

media supplemented with 10 mM maltose, melibiose, or glucose without or with 0.5 mM cAMP, and shaken at 37 °C. Absorption at 600 nm was monitored over 33 h. **(c)** Reconstitution of CyaA activity. *E. coli* DH5α or its *cya*A$^-$ strain transformed with one or two plasmids derived from either pCS19/X:T18 and pACYC/T25:X were plated on melibiose or maltose MacConkey agar plates supplemented with a selection antibiotic (100 mg/L ampicillin for the pCS19 derived plasmids and 35 mg/L chloramphenicol for pACYC derived plasmids) and 0.5 mM IPTG (inducer for protein expression), and incubated in 30 °C for 10 days. Red colonies were patched on fresh MacConkey agar plates under the same conditions.

sFig. 2. Supercomplex

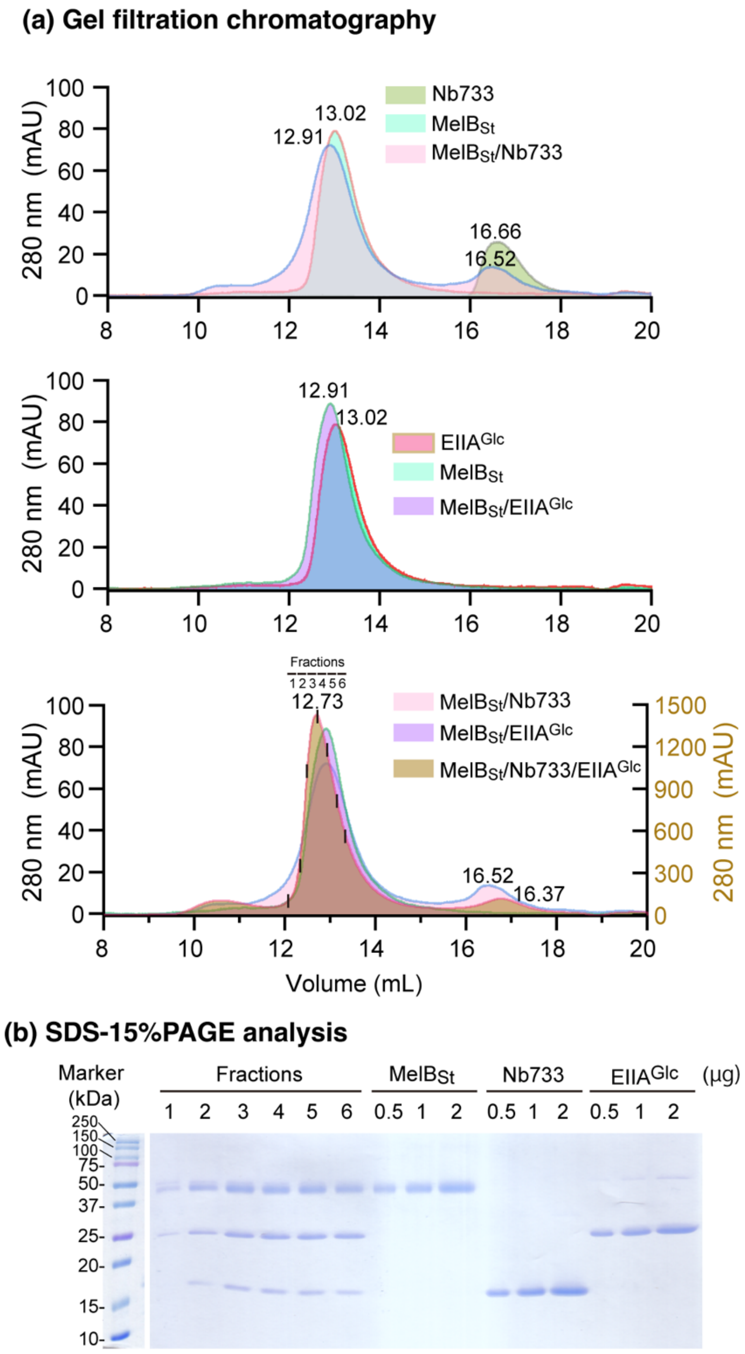

sFig. 2. Super-complex formation of MelB$_{St}$/Nb733/EIIA^{Glc}. (a) Gel-filtration chromatography. All three tests were performed with ENrich SEC 650 (Bio-Rad) pre-equilibrated with the sample buffer of 20 mM Tris-HCl, pH 7.5, 100 mM NaCl, 0.01% DDM and 10% glycerol on an NGC

Liquid Chromatography System (Bio-Rad) at room temperature. *Upper row,* $MelB_{St}$/Nb733 complex. $MelB_{St}$ alone (200 µg; filled in cyan), Nb733 alone (70 µg, filled in green), and the complex (filled in pink) at a molar ratio of 1: 1.2. *Middle row,* $MelB_{St}$/$EIIA^{Glc}$ complex. $EIIA^{Glc}$ alone (160 µg; filled in magenta) but no absorption at 280 nm with $EIIA^{Glc}$, and the $MelB_{St}$/$EIIA^{Glc}$ complex (filled in purple) at a molar ratio of 1:2. $MelB_{St}$ data were replotted from above. *Bottom row,* $MelB_{St}$/Nb733/$EIIA^{Glc}$ super-complex with the y-axe on the right side. $MelB_{St}$ (2 mg), Nb733 alone (700 µg), and $EIIA^{Glc}$ (1.6 mg) at a molar ratio of 1:1.2:2 (filled in dark yellow). The lines on the curve indicate the faction collection. The $MelB_{St}$/Nb733 and $MelB_{St}$/$EIIA^{Glc}$ complexes were replotted from above. **(b)** SDS-15%PAGE. 10 µL from each fraction as indicated, and 0.5, 1.0, and 2.0 µg of purified $MelB_{St}$, Nb733, or $EIIA^{Glc}$, were analyzed by SDS-15%PAGE and stained by Coomassie blue.
